# An impact assessment of the COVID-19 pandemic on Japanese and US hotel stocks

**DOI:** 10.1186/s40854-023-00478-2

**Published:** 2023-05-08

**Authors:** Takashi Kanamura

**Affiliations:** Graduate School of Advanced Integrated Studies in Human Survivability (GSAIS), Kyoto University, Kyoto, Japan

**Keywords:** Hotel industry, Asset price volatility, COVID-19, Regime-switching, Infection speed, C58, G01

## Abstract

This study proposes two new regime-switching volatility models to empirically analyze the impact of the COVID-19 pandemic on hotel stock prices in Japan compared with the US, taking into account the role of stock markets. The first model is a direct impact model of COVID-19 on hotel stock prices; the analysis finds that infection speed negatively affects Japanese hotel stock prices and shows that the regime continues to switch to high volatility in prices due to COVID-19 until September 2021, unlike US stock prices. The second model is a hybrid model with COVID-19 and stock market impacts on the hotel stock prices, which can remove the market impacts on regime-switching volatility; this analysis demonstrates that COVID-19 negatively affects hotel stock prices regardless of whether they are in Japan or the US. We also observe a transition to a high-volatility regime in hotel stock prices due to COVID-19 until around summer 2021 in both Japan and the US. These results suggest that COVID-19 is likely to affect hotel stock prices in general, except for the influence of the stock market. Considering the market influence, COVID-19 directly and/or indirectly affects Japanese hotel stocks through the Japanese stock market, and US hotel stocks have limited impacts from COVID-19 owing to the offset between the influence on hotel stocks and no effect on the stock market. Based on the results, investors and portfolio managers should be aware that the impact of COVID-19 on hotel stock returns depends on the balance between the direct and indirect effects, and varies from country to country and region to region.

## Introduction

The impact of the COVID-19 pandemic on tourism management has been enormous. For example, the number of foreign visitors to Japan in fiscal year (FY) 2013 was 10.4 million, which tripled to 31.9 million in FY2019, before the COVID-19 pandemic. However, by FY2020, after the spread of COVID-19, the number of foreign visitors had fallen to just 4.1 million, an 87.1% decline from FY2019. Japan is one of the world’s leading tourism countries, with a travel balance of $25 billion as of 2019, ranking 6th in the world (UNWTO 2020).^1^ Before the pandemic, expectations for the entire industry’s performance were growing, especially with the Tokyo 2020 Olympics and Paralympics Games (Tokyo 2020) scheduled to be held in July–September 2020, and the tourism industry making capital investments in preparation for these events. However, the International Olympic Committee postponed them for a year due to the global spread of COVID-19, and the Japanese government allowed only those involved in Tokyo 2020 to enter the country, making Tokyo 2020 an unprecedented event in which no spectators were allowed. In other words, it was difficult for Japan to balance the control of the COVID-19 pandemic with hosting the games in Tokyo in 2020. In this sense, the postponement significantly eroded investors’ expectations of Japan’s tourism industry. We consider that the impact of the COVID-19 pandemic on stock prices, expressed as expected values, was enormous for Japan, even as it aims to become a tourism nation (JNTO 2021) among the world’s major tourism nations.

Meanwhile, the US had the world’s largest travel balance at $62 billion in 2019 (UNWTO 2020), making it the largest tourism destination. While we expect that the impact of COVID-19 has been tremendous, the situation appears to differ from that in Japan. In the US, which implemented vaccination against COVID-19 relatively early in the pandemic, economic activities resumed earlier than in Japan after the spread of COVID-19. Thus, although the impact of COVID-19 on corporate activities in the tourism industry was huge, the situation differs from country to country, and we expect the impact of COVID-19 on stock prices to differ. The tourism industry includes a variety of sectors, specifically transport, accommodation, food and beverage, and entertainment. Of these, the sector that COVID-19 is likely to affect most is accommodation for long-stay tourism. Therefore, we focus on hotels, which are the main sector of the accommodation industry.

The aim of this study is to analyze the impact of COVID-19 on stock prices in the hotel industry, one of the sectors most affected by the pandemic. In particular, by comparing Japan, which we can consider a particularly serious situation due to the unprecedented disruption to the Olympic and Paralympic Games, with the US, which has the biggest tourist market but recovered early from the impact of the COVID-19 pandemic, we aim to highlight whether the impact differs from country to country. Specifically, we focus on the transition of the volatility in hotel stock prices due to COVID-19 to examine whether there are persistent impacts of COVID-19. Furthermore, we aim to provide implications for investors and portfolio managers. There are two research questions in this study. How has COVID-19 affected the stock prices of the Japanese hotel industry? How does this impact differ for hotels outside Japan, namely, in the US?

Research on the impact of COVID-19 on the financial and commodity markets is accelerating (Zaremba et al. 2020; Zhang et al. 2020; Dutta et al. 2020; Akhtaruzzaman et al. 2021; Ashraf 2020; Sharif et al. 2020; Xu 2021; Baig et al. 2021; Kanamura 2021; Athari et al. 2022; Athari and Hung 2022). These studies, in particular those using COVID-19 information explicitly or implicitly, provide meaningful and comprehensive examinations of the impact of COVID-19 on stock and commodity markets, but they do not provide a specific analysis of the travel and tourism industry, particularly the hotel industry, which is our aim.

Many researchers have studied the impact of exogenous risks on travel and tourism stocks (Demiralay and Kilincarslan 2019; García-Gómez et al. 2021; Yiwei et al. 2021; Carter et al. 2021; Irani et al. 2022).^2^ However, because these studies, except for pre-COVID-19 analyses of Demiralay and Kilincarslan (2019) and Irani et al. (2022), address the early stages of the COVID-19 pandemic, they do not directly address information about COVID-19-infected patients. Subsequently, many studies directly deal with COVID-19 information, such as the number of people infected with COVID-19, by examining the performance of hotel stocks under COVID-19 (Kaczmarek et al. 2021; Anguera-Torrell et al. 2021; Wu et al. 2021; Chen et al. 2020). These studies explicitly use COVID-19 information, including infection speed, to examine the impact of COVID-19 but do not model the regime changes in volatility that characterize the impact of COVID-19. While Lin and Falk (2021) and Baek et al. (2020) use regime-switching models, they do not use one in which COVID-19 directly affects returns.

Thus, there are gaps in the existing research that have yet to be filled. To the best of our knowledge, no analysis examines the direct impact of COVID-19 on price returns and regime-switching volatilities simultaneously; or investigates the impact of COVID-19 on the stock prices of the Japanese hotel industry. To fill these gaps, the present study compares Japanese hotel stocks with US ones and analyzes the impact of COVID-19 on these stocks using two new regime-switching volatility models whose returns are affected by the impact of the pandemic with and without stock market impacts. This analysis is significant in the sense that the new analytical approach may provide new insights into the impact of COVID-19 on hotel stock returns. In addition, a Japan–US comparison of the impact of COVID-19 on hotel stock prices offers hotel stock investment insights for investors and portfolio managers. This study adopts an econometric approach by using a reduced-type model called a regime-switching model. However, we incorporate the structure of stock market return impacts in the price returns of hotel stocks, along with variables representing the direct impact of COVID-19. In this sense, our analytical approach is a new mixed model between a reduced-type model that emphasizes data fitting and a structural-type model that emphasizes economic background.

This study makes three main contributions to the literature. First, it proposes two new regime-switching volatility models based on econometric analysis, in which the speed of COVID-19 infection directly affects price returns, and in which both COVID-19 and the stock market affect these returns. Second, using relatively long-term data from January 2019 to September 2021, we find that Japanese hotel stock returns have negative impacts from the infection speed in a direct and/or an indirect way via the corresponding market, while US hotel stock returns have a limited impact from COVID-19 due to the offset of the impact of COVID-19 on hotel stocks and no impact of COVID-19 on the overall stock market. Third, and most importantly, our analysis of Japanese hotel stock prices using a direct regime-switching volatility model shows that, unlike US hotel stock prices, the regime continues to switch to high volatility during COVID-19 until September 2021. However, analysis using the hybrid regime-switching volatility model, whose regime-switching excludes the impact of the stock market, shows a transition to high volatility in hotel stock prices due to the impact of COVID-19 until around the summer of 2021 in both Japan and the US.

The remainder of this paper is organized as follows. “Econometric analysis” section presents a simple econometric approach for determining the direction of our model’s selection of hotel stock returns. “The model” section proposes two new regime-switching volatility models, in which the COVID-19 infection speed directly affects price returns based on the results of the econometric analysis, and in which the COVID-19 infection speed and the market affect those. “Empirical analysis” section presents an empirical study of the differences in the impacts of COVID-19 on Japanese and US hotel stock returns. “Robustness checks” section presents the robustness examinations for our results. “Discussion of findings” section addresses the implications for investors, portfolio managers, tourism management, and policymakers with a comparative analysis with previous studies, an analysis of health and economic policies on COVID-19, policy recommendations, and financial flexibility and COVID-19. Finally, “Conclusions” section concludes and offers directions for future research.

## Econometric analysis

### Data

We use the Japanese hotel stock prices of Fujita Kanko, Inc. (FJT), Imperial Hotel, Ltd. (IMP), The Royal Hotel, Ltd. (RYL), The Kyoto Hotel, Ltd. (KYT), Oriental Land Co., Ltd. (OL), Seibu Holdings, Inc. (SB), and Kyoritsu Maintenance Co., Ltd. (KRT) and compare them with the US hotel stock prices of Choice Hotels International, Inc. (CHH), Hyatt Hotels Corporation (H), Hilton Worldwide Holdings, Inc. (HLT), InterContinental Hotels Group (IHG), Marriott International, Inc. (MAR), Wyndham Hotels & Resorts, Inc. (WH), and MGM Resorts International (MGM).^3^ In selecting hotel stocks, we take companies with large market capitalization (MC) as well as those with small MC. Specifically, in the case of Japanese hotel stocks, we select stocks ranging from OL, with an MC of 6,806.46 billion yen, to KYT, with an MC of only 7.40 billion yen, and in the case of US hotel stocks, from MAR, with an MC of 51.69 billion US dollars (USD), to CHH, with an MC of only 6.87 billion USD. In this way, the selection of stocks is based on the widest possible range of MC.^4^^,^^5^

**Fig. 1:**
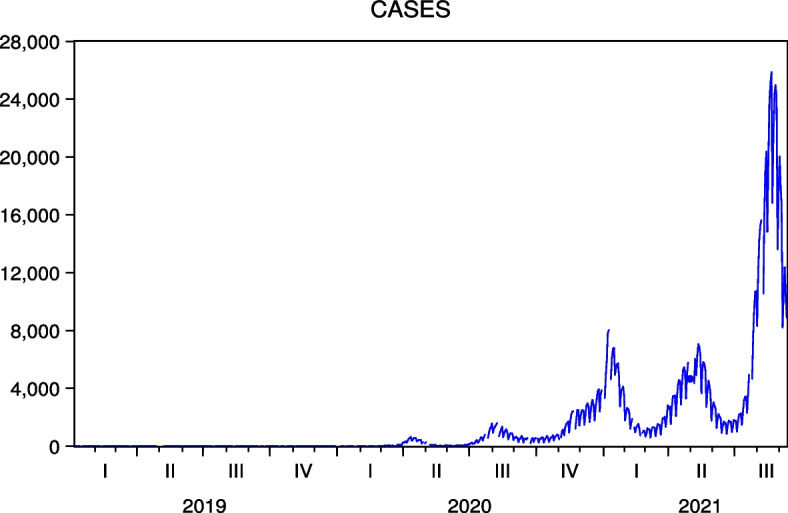
New COVID-19 Cases in Japan. Five waves are observed in Japan

**Fig. 2:**
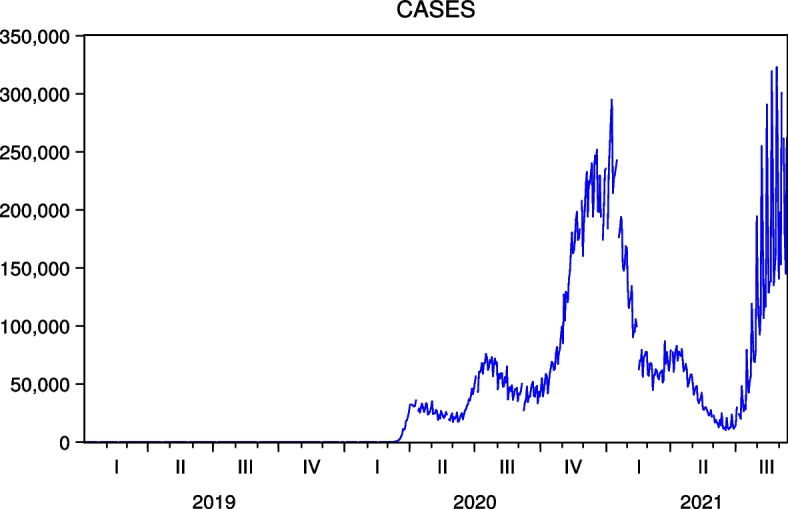
New COVID-19 Cases in the US. Five waves are observed in the US

The data cover January 2, 2019, to September 10, 2021, and are obtained from Investing.com.^6^ The period of the data strongly reflects the impact of COVID-19 on stock prices. Table 1 presents the basic data statistics.^7^ Note that we obtain COVID-19 cases in Japan and the US from the Ministry of Health, Labour and Welfare in Japan and Our World in Data, whose time series are shown in Figs. 1 and 2, respectively. We observe five waves of new COVID-19 infections in Japan and the US, respectively. In the case of Japan, the fifth wave was most pronounced in the summer of 2021, when the Tokyo 2020 games were held, while in the case of the US, the third and fifth waves were pronounced in the winter of 2020 to 2021 and the summer of 2021, respectively.

If there is a difference in the MC of Japanese and US hotel firms, it may affect the results of the analysis. To examine this point, we conduct a test of equality between the mean MC of Japanese and US hotel firms. To convert the Japanese hotel MC into US dollars, we employ the exchange rate of 110 USD/yen. The test results, shown in Table  1, confirm that there is no difference in the mean MC of Japanese and US hotel companies, and thus, do not reject the null hypothesis of equal means.

**Table 1:** Basic statistics of Japan and US hotel stock prices and test of equality of means of market capitalization between Japan and US hotels

	FJT	IMP	RYL	KYT	OL	SB	KRT
Mean	2202.723	1927.335	1441.844	665.536	14,864.080	1478.145	4140.329
Std. Dev.	542.943	114.232	237.174	79.436	1663.452	333.170	835.310
Skewness	− 0.105	− 2.499	0.307	0.074	− 0.261	0.138	− 0.047
Kurtosis	1.411	11.866	1.363	1.489	2.561	1.409	2.537
Market Cap.	30.14	107.04	12.27	7.40	6,806.46	450.26	187.08

	CHH	H	HLT	IHG	MAR	WH	MGM
Mean	94.014	70.976	98.000	60.602	122.866	55.928	28.594
Std. Dev.	14.823	11.492	17.875	8.485	21.335	10.455	8.084
Skewness	0.098	− 0.631	0.119	− 1.022	− 0.728	− 0.128	− 0.089
Kurtosis	2.394	2.535	2.063	3.688	2.474	3.264	2.530
Market Cap.	6.87	9.09	38.55	11.21	51.69	6.93	14.31

Method	df	Value	Probability				
t-test	12	− 0.903	0.384				
Satterthwaite-Welch t-test^a^	11.319	− 0.903	0.385				
Anova F-test	(1, 12)	0.815	0.384				
Welch F-test^a^	(1, 11.320)	0.815	0.385				

Note that market capitalization (MC) for Japanese hotels is in Billion Yen, which is obtained from the website of the Nikkei as of May 2022 and that for the US hotels is in Billion USD, which is obtained from the website of Companiesmarketcap.com as of May 2022. Note also the exchange rate is 110 USD/yen for a test of equality of means of MC between Japan and US hotels. The test results confirm that there is no difference in the mean MC of Japanese and US hotel companies, not rejecting the null hypothesis of equal means

^a^Test allows for unequal cell variances

### Analyses

To justify the price–return models proposed in this study, we perform unit root with break tests for logarithmic prices using Zivot and Andrews (2002) and its application (e.g., Kirikkaleli and Athari 2020) assuming the trend specification is trend and intercept, and the break specification is trend only, as an example. Table 2 suggests that almost all log asset prices except for IMP have a unit root. In addition, in Table 2, for LIMP, LKYT, and LKRT in Japan and LCHH, LH, LIHG, LMAR, LWH, and LMGM in the US, we confirm a structural break around late March, at the start of the COVID-19 pandemic. This results in the possibility of introducing log-price differences that have a random walk with constant drift and regime-switching.^8^

**Table 2:** Unit root with break tests for log prices

Japanese Hotels	LFJT		LIMP		LRYL		LKYT
Zivot-	t-Stat	Pr.	t-Stat	Pr.	t-Stat	Pr.	t-Stat
Andrews	− 2.379	0.946	− 4.658	0.035	− 3.666	0.319	− 2.345
	Break:	Pr.	Break:	Pr.	Break:	Pr.	Break:
	7/31/'20	0.008	3/17/'20	0.042	11/06/'20	0.002	4/06/'20

US Hotels	LCHH		LH		LHLT		LIHG
Zivot-	t-Stat	Pr.	t-Stat	Pr.	t-Stat	Pr.	t-Stat
Andrews	− 3.324	0.512	− 3.229	0.572	− 2.020	0.989	− 2.583
	Break:	Pr.	Break:	Pr.	Break:	Pr.	Break:
	4/01/'20	0.021	5/13/'20	0.019	1/08/'19	0.146	3/17/'20

Japanese Hotels		LOL		LSB		LKRT	
Zivot-	Pr.	t-Stat	Pr.	t-Stat	Pr.	t-Stat	Pr.
Andrews	0.953	− 3.646	0.328	− 3.152	0.618	− 2.348	0.952
	Pr.	Break:	Pr.	Break:	Pr.	Break:	Pr.
	0.010	7/30/'19	0.099	11/27/'20	0.006	3/16/'20	0.038

US Hotels		LMAR		LWH		LMGM	
Zivot-	Pr.	t-Stat	Pr.	t-Stat	Pr.	t-Stat	Pr.
Andrews	0.890	− 3.144	0.622	− 2.737	0.829	− 2.280	0.964
	Pr.	Break:	Pr.	Break:	Pr.	Break:	Pr.
	0.025	5/05/'20	0.010	3/18/'20	0.015	5/12/'20	0.024

Null hypothesis: log prices have a unit root; exogenous: a constant and trend; trend break; almost all log asset prices except for IMP have a unit root

To investigate the effect of COVID-19 on hotel stock returns, we conduct a Granger causality test on the speed of infection and logarithm returns of hotel stock prices. Table 3 shows the results for the stock prices of Japanese hotels (FJT, IMP, RYL, KYT, OL, SB, and KRT) and US hotels (CHH, H, IHG, HLT, MAR, WH, and MGM). They report that the logarithmic price returns of FJT, SB, and KRT for Japanese companies are Granger-caused by the speed of increase in the number of infections at significance levels of 9%, 7%, and 2%, respectively, whereas the logarithmic price returns for US hotel companies are not Granger-caused at all by the speed of increase in the number of infections. Thus, these results suggest that we can model the Japanese hotel industry’s stock returns affected by the speed of infection growth in Japan, while we can model the US returns unaffected by the speed of infection growth in the US.^9^ Note that according to Table 1, FJT and KYT are relatively small firms in terms of MC, while SB is a medium-sized firm. However, we cannot capture Granger causality for OL with large MC. In this sense, we are able to capture Granger causality as a preliminary analysis for small and medium-sized Japanese hotel companies.

**Table 3:** Granger causality test Lag = 2

Null hypothesis	Obs	F-Statistic	Prob.
DCASES does not Granger Cause LRFJT	552	2.37	0.09
DCASES does not Granger Cause LRIMP	552	1.11	0.33
DCASES does not Granger Cause LRRYL	552	0.26	0.77
DCASES does not Granger Cause LRKYT	552	0.77	0.46
DCASES does not Granger Cause LROL	552	0.80	0.45
DCASES does not Granger Cause LRSB	552	2.69	0.07
DCASES does not Granger Cause LRKRT	552	3.95	0.02
DCASES does not Granger Cause LRCHH	604	0.12	0.89
DCASES does not Granger Cause LRH	604	0.82	0.44
DCASES does not Granger Cause LRHLT	604	0.48	0.62
DCASES does not Granger Cause LRIHG	604	0.38	0.68
DCASES does not Granger Cause LRMAR	604	0.15	0.86
DCASES does not Granger Cause LRWH	604	0.47	0.62
DCASES does not Granger Cause LRMGM	604	0.28	0.76

The logarithmic price returns of FJT, SB, and KRT for Japanese companies were found to be Granger-caused by the speed of increase in the number of infections at the significance levels of 9%, 7%, and 2%, respectively

Based on the above discussion, it is necessary to construct a model that can confirm that changes in the number of COVID-19 cases in Japan affect Japanese hotel stock returns, whereas those in the US do not affect the US hotel stock returns. The next section proposes such a model in which the statistical significance of the model parameter estimates can determine the impact of the changes.

## The model

It is generally known in economics that non-diversifiable risk (i.e., systematic risk) is the only risk necessary for stock valuation. As we consider the risk of COVID-19 as an undiversifiable risk inevitable in corporate efforts, this study incorporates the impact of COVID-19 into the model. In addition, the results of the preliminary empirical analysis in “Econometric analysis” section suggest that we can model hotel stock returns in Japan as influenced by the change in the number of infected people one period earlier. These points are why we incorporate the change in the number of infections one period before as the explanatory variable for stock returns, which is in line with the literature on tourism stocks (Kaczmarek et al. 2021; Anguera-Torrell et al. 2021; Wu et al. 2021).^10^ In addition, existing studies on financial and commodity markets (e.g., Baek et al. 2020; Kanamura 2021), particularly tourism stocks (Lin and Falk 2021; Baek et al. 2020), show regime-switching of price volatility due to the impact of COVID-19. First, we propose a new regime-switching volatility model in which the COVID-19 infection speed directly affects log price returns ($\log S_{t+1}- \log S_{t}$) based on empirical analyses.^11^

1 $$\begin{aligned} \log S_{t+1}- \log S_{t}=\left\{ \begin{array}{l} C_{1} +\lambda _1 \Delta \text {Cases}_t+\exp (k_1)\epsilon _t, \\ C_{2} +\lambda _2 \Delta \text {Cases}_t+\exp (k_2)\omega _t. \end{array} \right. \end{aligned}$$

Note that $\Delta \text {Cases}_t=\text {Cases}_t-\text {Cases}_{t-1}$, the changes in new COVID-19 cases, represent COVID-19 infection speed, and $\exp (k_1)$ and $\exp (k_2)$ represent the corresponding regime volatilities. We employ constant transition probabilities. $p_{ij}$ represents the transition probability from state *i* at time $t-1$ to state *j* at time *t*.

2 $$\begin{aligned} P= \begin{pmatrix} p_{11} &{} p_{12} \\ p_{21} &{} p_{22} \end{pmatrix}&= \begin{pmatrix} p_{11} &{} 1-p_{11} \\ p_{21} &{} 1-p_{21} \end{pmatrix} \end{aligned}$$

3 $$\begin{aligned} p_{11}&=\frac{\exp (\delta _1)}{1+\exp (\delta _1)} \end{aligned}$$

4 $$\begin{aligned} p_{21}&=\frac{\exp (\delta _2)}{1+\exp (\delta _2)} \end{aligned}$$

Eqs. (1) to (4) represent a new direct model with the regime-switching volatility of assets whose returns directly depend on COVID-19 infection speed and, to the best of our knowledge, have not been used in existing studies on the impact of COVID-19 on asset price volatility (e.g., Ashraf 2020; Kanamura 2021), particularly on hotel stock price volatility.

The direct impact of the changes in the number of COVID-19 cases on hotel stock prices may propagate through the market. Second, we propose a new hybrid model of hotel stock prices that includes both market impact and changes in the number of COVID-19 cases.

5 $$\begin{aligned} \log S_{t+1}- \log S_{t}=\beta (\log S^M_{t+1}- \log S^M_{t})+\left\{ \begin{array}{l} C_1+\lambda _1 \Delta \text {Cases}_t+\exp (k_1)\epsilon _t, \\ C_2+\lambda _2 \Delta \text {Cases}_t+\exp (k_2)\omega _t. \end{array} \right. \end{aligned}$$

The market log price return ($\log S^M_{t+1}- \log S^M_{t}$) represents the market impact. The two new models proposed in Eqs. (1) and (5) are the first contribution of this study.

## Empirical analysis

### Results

This section investigates the impact of the COVID-19 pandemic on hotel stock returns, based on the parameter estimation results of a direct impact model of COVID-19 in Eqs. (1) to (4). Table 4 reports the results of Japanese hotel stocks. We assume zero constants in Eq. (1), because the preliminary empirical analyses suggest no statistical significance of the coefficients. In all model parameter estimation results for the Japanese hotel stocks of FJT, IMP, RYL, KYT, OL, SB, and KRT in Table 4, the different value estimates of $k_1$ and $k_2$, two regime volatilities, and those of $\delta _{1}$ and $\delta _{2}$, and the transition probabilities from Regime 1 to Regime 1 and Regime 2 to Regime 1, respectively, are statistically significant, indicating that there are two explicit regimes with different volatilities and that regime-switching occurs. In particular, for IMP, RYL, KYT, SB, and KRT, $k_1<k_2$ indicates that Regime 2 is a high-volatility regime and Regime 1 is a low-volatility regime, whereas for FJT and OL, $k_1>k_2$ indicates that Regime 1 is a high-volatility regime and Regime 2 is a low-volatility regime.

We also find that $\lambda _1$s of RYL, KYT, OL, and SB and $\lambda _2$s of FJT and KRT in Table 4, representing the impact of COVID-19 infection speed on stock price returns in Regimes 1 and 2, respectively, are statistically significant negative estimates, while $\lambda _1$ of KYT is weakly statistically significant. Negative impacts occurred in low-volatility regimes for FJT, RYL, KYT, and SB, and in high-volatility regimes for OL and KRT. Thus, the impact of the infection speed of COVID-19 on price returns manifests as a lowering effect on price returns in a low- or high-volatility regime. The preliminary test in Table 3 partially predicts this result. In particular, we confirm the stock return reduction effect due to COVID-19 for OL with a large firm size, which means that we confirm the effect of COVID-19 on stock return reduction regardless of firm size. The results are consistent with those of Wu et al. (2021) in Chinese markets. We find that the speed of COVID-19 infection in the high- or low-volatility regime negatively impacts Japanese hotel stock returns when we use long-term data from January 4, 2019, to September 10, 2021. In addition, this result indicates a negative impact of COVID-19 on price returns across the sample over an 18-month period following the spread of COVID-19. In other words, the impact of COVID-19 is long-lasting.

**Table 4:** Direct model parameter estimation for Japanese hotel stocks

	Regime 1		Regime 2		T-Matrix		LL	AIC	SIC
	*λ*_1_	*k*_1_	*λ*_2_	*k*_2_	*δ*_1_	*δ*_2_			
FJT	− 9.520E−07	− **3.438**	− **1.730**E−**06**	− **4.281**	**3.400**	− **4.053**	1464	− 2916	− 2889
IMP	3.690E−07	− **4.956**	3.700E−08	− **3.205**	**3.795**	− **1.805**	1876	− 3739	− 3713
RYL	− **4.920**E−**07**	− **5.252**	3.750E−07	− **3.742**	**2.921**	− **2.485**	1811	− 3611	− 3584
KYT	− *4.680*E−*07*	− **5.172**	1.800E−08	− **3.685**	**3.256**	− **2.644**	1805	− 3597	− 3571
OL	− **6.470**E−**05**	− **3.515**	− 6.840E−07	− **4.336**	**2.333**	− **4.453**	1626	− 3241	− 3214
SB	− **2.050**E−**06**	− **4.374**	− 8.950E−07	− **3.538**	**4.381**	− **4.095**	1488	− 2964	− 2938
KRT	− 9.070E−07	− **4.108**	− **1.470**E−**05**	− **3.304**	**4.030**	− **3.296**	1377	− 2742	− 2716

Note that the bold and italic numbers represent statistical significance and weakly statistical significance at 5$\%$ and 10$\%$, respectively. $\lambda _1$s of RYL, KYT, OL, and SB, and $\lambda _2$s of FJT and KRT in Table 4, representing the impact of COVID-19 infection speed to stock price returns in Regimes 1 and Regimes 2, respectively, are statistically significant negative estimates, while $\lambda _1$ of KYT is weakly statistically significant

**Fig. 3:**
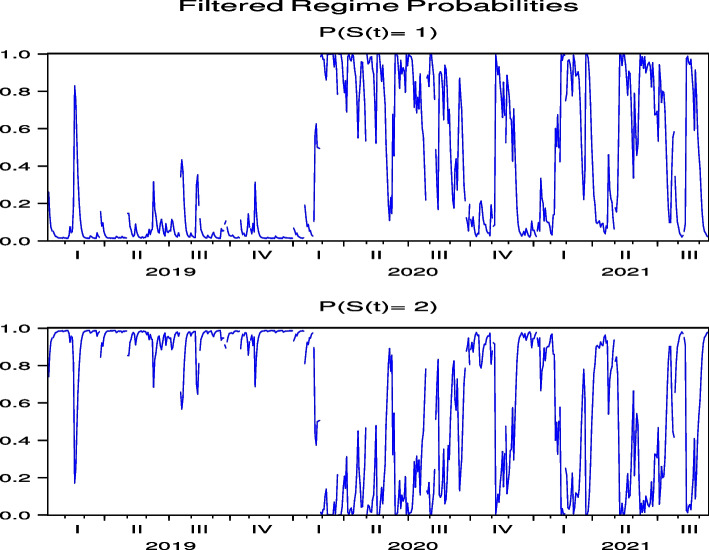
Regime probabilities for FJT. After February 2020, the regime probabilities moved into a regime of high price volatility, then moved into a regime of low one until after the 2021 summer

**Fig. 4:**
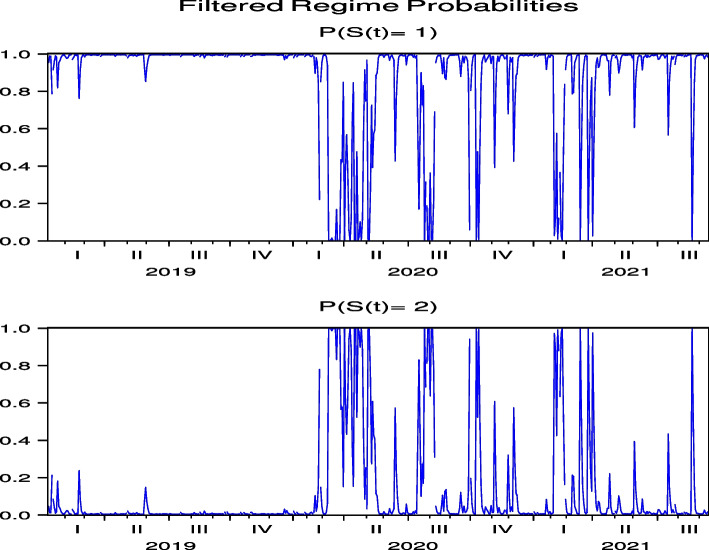
Regime probabilities for IMP. After February 2020, the regime probabilities moved into a regime of high price volatility, then moved into a regime of low one until almost after the 2021 summer

Next, we consider the regime probabilities based on the parameter estimation results.^12^ Figures 3 and 4, which report the regime probabilities of FJT and IMP, respectively, show that, following the global spread of COVID-19 in February 2020, the regime probabilities moved into a regime of high price volatility and then into a regime of low price volatility, where we observe convergence. Figures 19, 20, 21, 22 and 23 for RYL, KYT, OL, SB, and KRT in “Appendix 1”, respectively, offer the same results of regime probabilities to those of FJT and IMP. This is consistent with the parameter estimation in Table 4, because Regime 2 is a high-volatility regime for IMP, RYL, KYT, SB, and KRT, and Regime 1 is a high-volatility regime for FJT and OL. However, except for OL, the regime probabilities then return to the high-volatility regime until after the summer of 2021.^13^ This indicates that the regime switch phenomenon of Japanese hotel stocks almost repeats until after the summer of 2021.

**Fig. 5:**
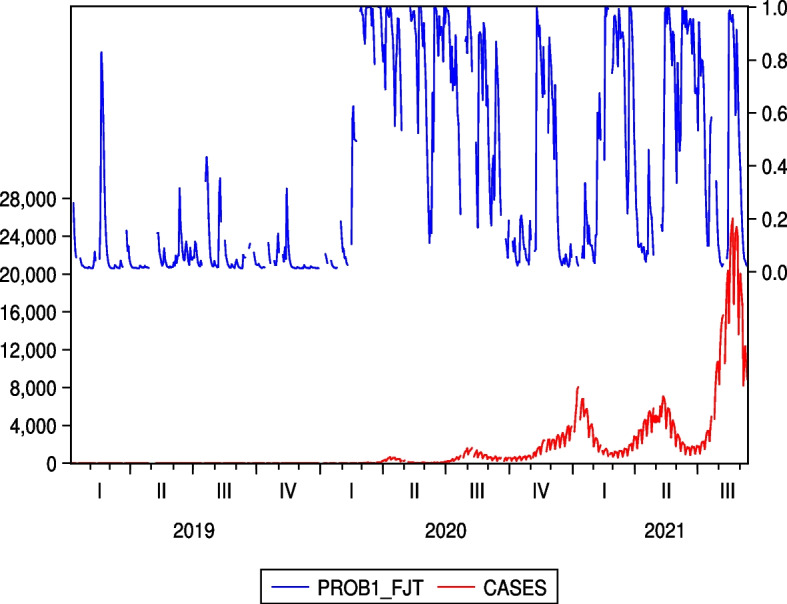
Regime probabilities for FJT and COVID-19 cases. High volatility regime probabilities tend to correspond to the peaks of the cases

**Fig. 6:**
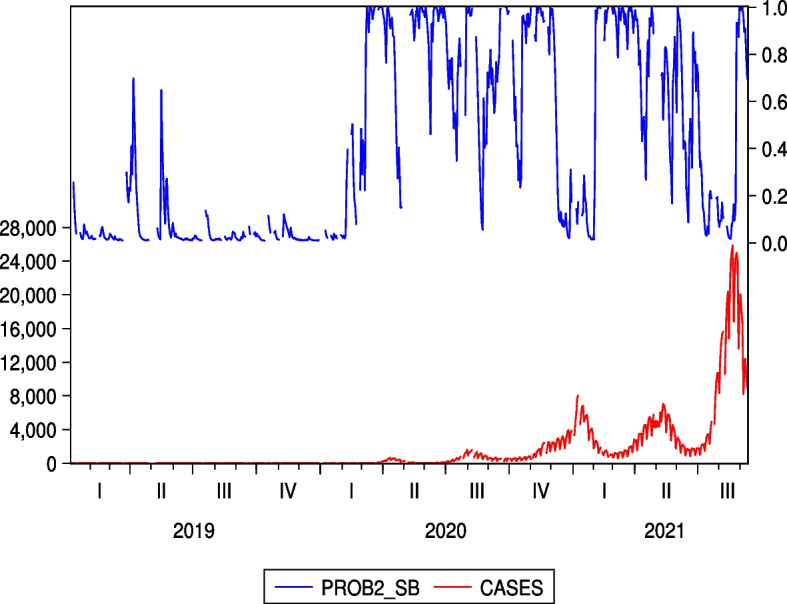
Regime probabilities for SB and COVID-19 cases. High volatility regime probabilities tend to correspond to the peaks of the cases, similar to FJT

There have been five waves of COVID-19 infections in Japan, as shown in Fig. 1. As the speed of infection negatively affects price returns, we can expect the regime probabilities to follow these five waves in any form. To highlight this point, we show the relationship between the high-volatility regime probabilities and COVID-19 cases in the same picture for FJT and SB in Figs. 5 and 6, respectively, which have statistically significant negative impacts of COVID-19 infection speed on stock price returns. We observe that the high-volatility regime probabilities tend to correspond to the peaks of these cases. More importantly, the impact of COVID-19 on Japanese hotel stock prices persists even at the peak of the fifth wave in August 2021.

To compare the results using Japanese hotel stocks, we investigate the impact of COVID-19 on US hotel stock returns based on the estimation results of the model parameters. We also assume zero constants for the model because of the insignificance. All the estimation results in Table 5 for CHH, H, HLT, IHG, MAR, WH, and MGM indicate, similar to Japanese hotels, that there are two explicit regimes with different volatilities and that regime-switching occurs. Note that $k_1>k_2$ for CHH, H, MAR, and WH indicates that Regime 1 is a high-volatility regime and Regime 2 is a low-volatility regime, whereas $k_1<k_2$ for HLT, IHG, and MGM indicates that Regime 2 is a high-volatility regime and Regime 1 is a low-volatility regime.^14^

However, the impact of the infection speed of COVID-19 on US price returns is quite different from the Japanese case. The parameters $\lambda _1$ and $\lambda _2$, which represent the impact of the speed of infection of COVID-19 on price returns in Regimes 1 and 2, respectively, are not statistically significant in Table 5, resulting in no impact of the COVID-19 infection speed on price returns in Regimes 1 and 2. Thus, we find that the speed of COVID-19 infection in Japan negatively impacts Japanese hotel stock returns, while US hotel stock returns are perfectly insensitive to the speed of COVID-19 infection in the US when using relatively long-term data from January 2019 to September 2021.

**Table 5:** Direct model parameter estimation for US hotel stocks

	Regime 1		Regime 2		T-Matrix		LL	AIC	SIC
	*λ*_1_	*k*_1_	*λ*_2_	*k*_2_	*δ*_1_	*δ*_2_			
CHH	1.340E−07	− **3**.**193**	− 2.090E−08	− **4**.**401**	**3**.**143**	− **4**.**353**	1651	− 3290	− 3264
H	− 1.670E−07	− **2**.**961**	− 1.400E−08	− **4**.**217**	**2**.**526**	− **3**.**718**	1506	− 2999	− 2973
HLT	− 2.900E− 08	− **4**.**222**	1.330E−08	− **3**.**134**	**3**.**820**	− **2**.**343**	1579	− 3146	− 3119
IHG	− 3.650E−08	− **4**.**151**	− 5.260E−07	− **2**.**849**	**5**.**221**	− **3**.**383**	1572	− 3132	− 3106
MAR	− 4.560E−08	− **2**.**923**	− 1.050E−08	− **4**.**132**	**3**.**263**	− **4**.**536**	1505	− 2999	− 2972
WH	− 2.280E−07	− **2**.**715**	− 3.050E−08	− **4**.**079**	**2**.**812**	− **4**.**695**	1521	− 3030	− 3003
MGM	− 4.590E−08	− **3**.**865**	− 4.310E−08	− **2**.**466**	**4.640**	− **3**.**025**	1343	− 2673	− 2646

Note that the bold numbers represent statistical significance at 5$\%$. The parameters $\lambda _1$ and $\lambda _2$, which represent the impact of the speed of infection of COVID-19 on price returns in Regimes 1 and 2, respectively, are not statistically significant

**Fig. 7:**
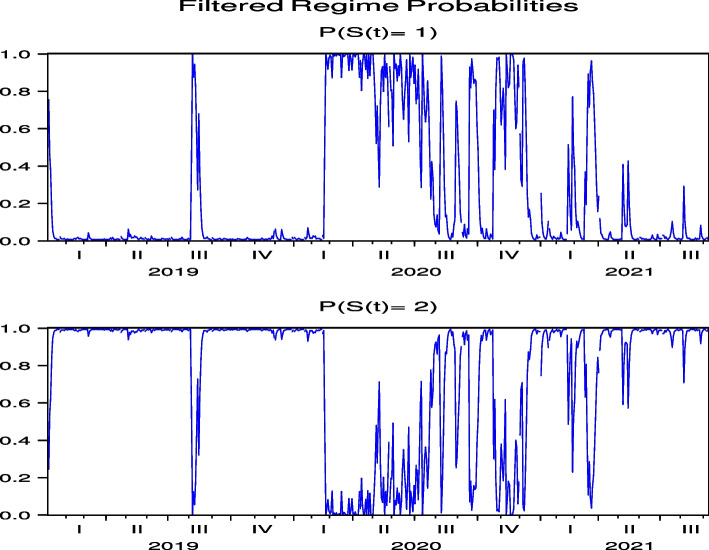
Regime probabilities for CHH. After February 2020, the regime probabilities shifted to a regime with high price volatility, and then converged to a lower volatility regime soon

**Fig. 8:**
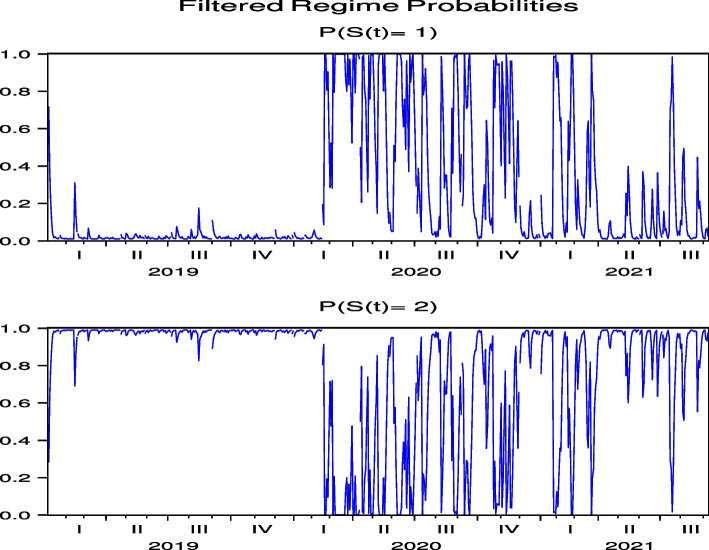
Regime probabilities for H. After February 2020, the regime probabilities shifted to a regime with high price volatility, and then converged to a lower volatility regime over time

Next, we consider the regime probabilities based on the parameter estimation results. The regime probabilities from Figs. 7 and 8 for CHH and H, respectively, show that with the global spread of COVID-19 in February 2020, the regime probabilities shifted to a regime with high price volatility and then converged to a lower-volatility regime. Figures 24, 25, 26, 27 and 28 for HLT, IHG, MAR, WH, and MGM in “Appendix 1”, respectively, offer the same regime probability results for CHH and H. This result is consistent with the parameter estimation in Table 5, because Regime 2 is a high-volatility regime for HLT, IHG, and MGM, and Regime 1 is a high-volatility regime for CHH, H, MAR, and WH. However, unlike the case of Japan, in the US, the subsequent switch back to the high-volatility regime weakened over time, except for H, indicating that the regime-switching phenomenon had largely subsided by September 2021. Thus, our analysis of Japanese hotel stock prices shows that, unlike US hotel stock prices, high-frequency regime-switching to high volatility due to COVID-19 persisted until September 2021, when 1.5 years had passed since the first COVID-19 outbreak.

**Fig. 9:**
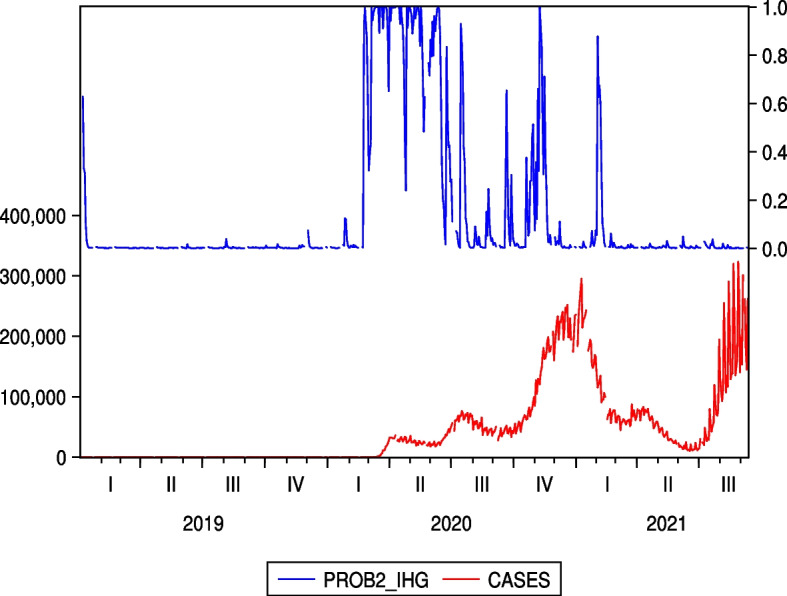
Regime probabilities for IHG and COVID-19 cases. The figure did not show an increase in regime probability associated with a sharp increase in the number of infected people in the third quarter of 2021

**Fig. 10:**
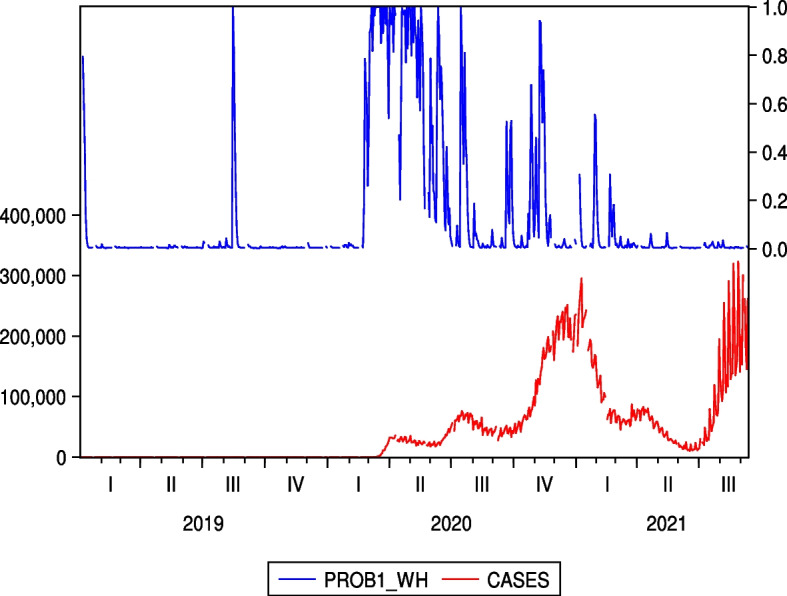
Regime probabilities for WH and COVID-19 cases. The figure did not show an increase in regime probability associated with a sharp increase in the number of infected people in the third quarter of 2021, similar to IHG

Unlike Figs. 5 and 6, which are two examples of the Japanese hotel industry, Figs. 9 and 10, which are the cases of IHG and WH as two examples of the US hotel industry, do not show an increase in regime probability associated with a sharp increase in the number of infected people in the third quarter of 2021. From this point of view, the results of Figs. 5 and 6, shown as two examples for the Japanese hotel industry, are unique and illustrate the continued impact of COVID-19 on Japanese hotel stock prices.

### Stock market and COVID-19

**Fig. 11:**
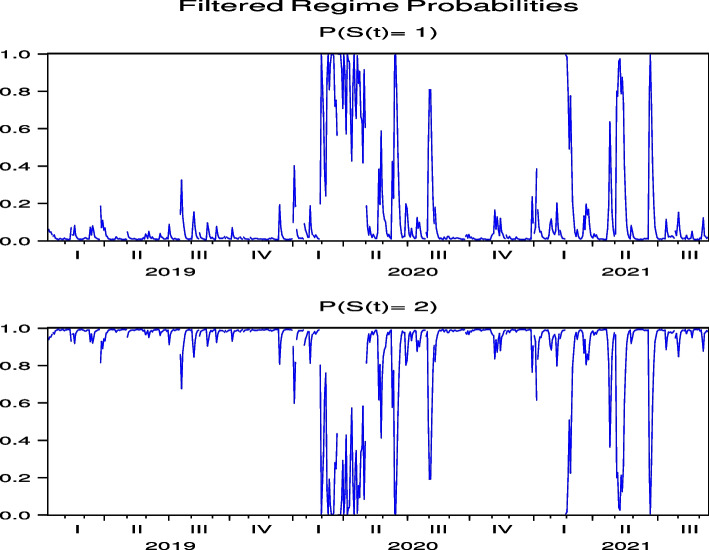
Regime probabilities for NKY. The impact of COVID-19 on NKY continues until summer of 2021 while weaker than Japanese hotel stocks

**Fig. 12:**
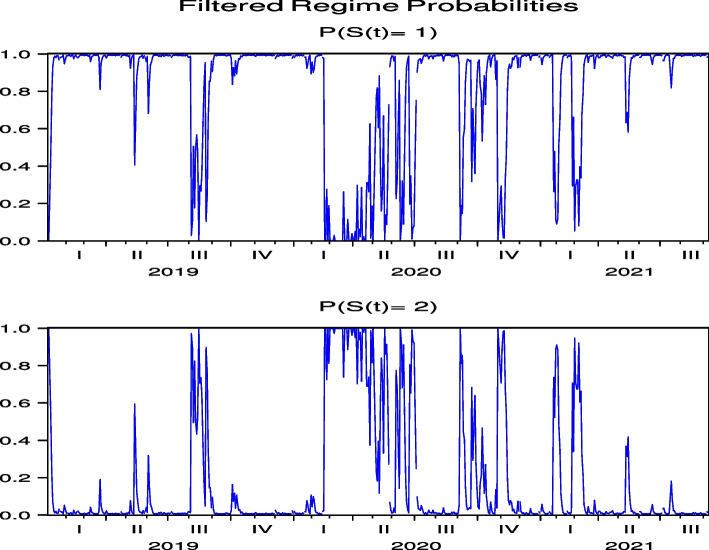
Regime probabilities for SPX. The impact of COVID-19 on SPX is relatively short-lived, which is the same as the case of US hotel stock prices

We examine the direct impact of COVID-19 on hotel stocks; however, because the overall stock market influences hotel stock prices, it is important to examine the relationship between hotel stock prices, stock markets, and COVID-19. Therefore, we first perform an empirical analysis using the previous model in Eq. (1) with stock indexes, and Table 6 shows the results for the Nikkei 225 (NKY) and S&P 500 (SPX). Similar to Japanese and US hotel stocks, the model parameter estimation results for NKY and SPX indicate that there are two explicit regimes with different volatilities and that regime-switching occurs, where Regimes 1 and 2 have high-volatility regimes for NKY and SPX, respectively. We also find that $\lambda _2$ of NKY in Table 6, representing the impact of COVID-19 infection speed on stock price returns in Regime 2, is a statistically significant negative estimate, while both $\lambda _1$ and $\lambda _2$ of SPX are not statistically significant. Thus, the impact of the speed of infection of COVID-19 on NKY price returns manifests as a lowering effect on price returns in a low-volatility regime, while we do not find this result for SPX price returns. Similar to the results for Japanese hotel stock prices, COVID-19 directly affects the Japanese stock index, but does not directly affect the US stock index. Thus, COVID-19 can indirectly affect hotel stock prices in Japan through the overall stock market, and the US stock market can alleviate the impact of COVID-19 on hotel stock prices in the US. The regime probabilities in Fig. 11 show that the impact of COVID-19 on NKY continued until the summer of 2021, while the continuous impact was weaker than the impact on Japanese hotel stocks, except for OL. The regime probabilities in Fig. 12 show that the impact of COVID-19 on SPX is relatively short-lived, which is the same as the case for US hotel stock prices, except for H.

**Table 6:** Direct model parameter estimation for NKY and SPX

	Regime 1		Regime 2		T-Matrix		LL	AIC	SIC
	*λ*_1_	*k*_1_	*λ*_2_	*k*_2_	*δ*_1_	*δ*_2_			
NKY	− 4.110E−06	− **3.652**	− **7.420**E−**07**	− **4.716**	**2.111**	− **4.243**	1377	− 2742	− 2716
SPX	1.600E−08	− **4.936**	− 5.600E-08	− **3.449**	**3.919**	− **2.323**	1992	− 3973	− 3946

Note that the bold numbers represent statistical significance at 5$\%$. $\lambda _2$ of NKY, representing the impact of COVID-19 infection speed to stock price returns in Regime 2 is statistically significant negative estimates, while both $\lambda _1$ and $\lambda _2$ of SPX are not statistically significant

To test this market-impact structure and examine the impact of COVID-19 on hotel stocks, we second estimate a new hybrid model in Eq. (5) using the rate of change in the stock price index as a control variable. We assume zero constants for the model in Eq. (5), because of the insignificance. The estimation results for Japanese hotel stocks in Table 7 show that $\beta$s, representing the sensitivity of the market to hotel stock prices, are statistically significant, implying that the stock market strongly affects Japanese hotel stock returns. $\lambda _2$s for SB and KRT, representing the impact of changes in the number of COVID-19 cases on hotel stock prices, have strongly statistically significant negative values, and $\lambda _2$ for RYL has a weakly statistically significant negative value. Thus, taken together with the results for the stock market impacted by the COVID-19 infection number change, that is, the total number of statistically significant negative $\lambda _1$ and $\lambda _2$ estimates for the model parameter estimation in Eq. (5) is less than that in Eq. (1), as shown in Table 4, it is likely that the negative impacts of the changes in COVID-19 infection cases on Japanese hotel stock prices indirectly come from the stock market. Additionally, even if we remove the impact of the Japanese stock market on Japanese hotel stocks, we find a negative impact of COVID-19 case changes on Japanese hotel stock prices.

**Table 7:** Hybrid model parameter estimation for Japanese hotel stocks

	Regime 1		Regime 2		Constant	T-Matrix		LL	AIC	SIC
	*λ*_1_	*k*_1_	*λ*_2_	*k*_2_	*β*	*δ*_1_	*δ*_2_			
FJT	− 6.810E−07	− **3.707**	− 8.150E−07	− **4.530**	**0.858**	**4.197**	− **4.252**	1542	− 3070	− 3039
IMP	6.100E−07	− **3.289**	**5.500**E−**07**	− **5.016**	**0.225**	**1.842**	− **3.762**	1903	− 3792	− 3761
RYL	7.440E−07	− **3.770**	− *4.170*E−*07*	− **5.226**	**0.138**	**2.502**	− **3.009**	1820	− 3626	− 3596
KYT	2.320E−07	− **3.712**	− 3.850E−07	− **5.178**	**0.137**	**2.606**	− **3.225**	1813	− 3611	− 3581
OL	1.930E−09	− **3.685**	− 3.350E−07	− **4.498**	**0.553**	**1.814**	− **3.469**	1683	− 3352	− 3322
SB	1.020E−07	− **3.597**	− **1.780**E−**06**	− **4.428**	**0.719**	**3.519**	− **4.071**	1548	− 3082	− 3051
KRT	− 1.590E−07	− **4.254**	− **8.400**E−**06**	− **3.542**	**0.992**	**4.249**	− **3.776**	1459	− 2904	− 2874

Note that the bold and italic numbers represent statistical significance and weakly statistical significance at 5$\%$ and 10$\%$, respectively. $\beta$s, representing the sensitivity of the market to hotel stock prices, are statistically significant. $\lambda _2$s for SB and KRT, representing the impact of changes in the number of COVID-19 cases on hotel stock prices in Regime 2, are strongly statistically significant negative values and $\lambda _2$ for RYL is a weakly statistically significant negative value

**Fig. 13:**
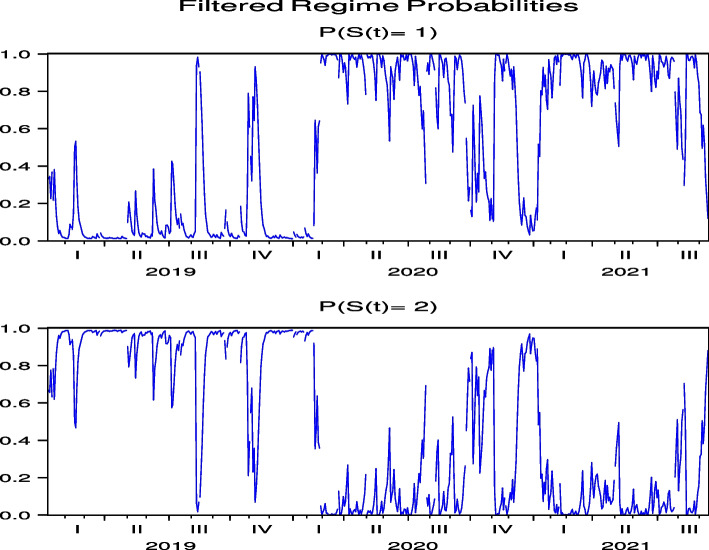
Regime probabilities for FJT. The transition to the high volatility regime continues through after summer of 2021

**Fig. 14:**
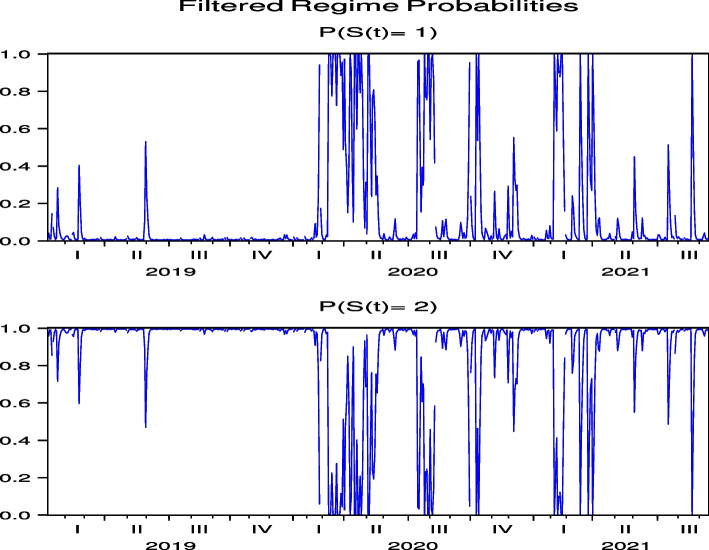
Regime probabilities for IMP. The transition to the high volatility regime continues through after summer of 2021, similar to FJT

The regime probabilities in Figs. 13 and 14, obtained from the hybrid model using the Japanese hotel stock returns of FJT and IMP, respectively, show that the transition to the high-volatility regime continues until after the summer of 2021. Note that Figs. 29, 30, 31, 32 and 33 for RYL, KYT, OL, SB, and KRT in “Appendix 1”, respectively, offer the same results for the regime probabilities as FJT and IMP, except for OL. This result is consistent with the results of the model analysis, including the direct impact of COVID-19.

The estimation results for US hotel stocks in Table 8 show that $\beta$s, representing the sensitivity of the market to hotel stock prices, are statistically significant, implying that the stock market strongly affects US hotel stock returns. $\lambda _2$ for MGM, which represents the impact of changes in the number of COVID-19 cases on hotel stock prices, has a statistically significant negative value. $\lambda _1$ values for CHH, H, IHG, and WH have weak but statistically significant negative values. The results differ from those with only no impact of COVID-19 on US hotel stock prices in Table 5. This result suggests that COVID-19 has a negative impact on US hotel stocks, like Japanese hotel stocks, but the impacts are offset by the market.

**Table 8:** Hybrid model parameter estimation for US hotel stocks

	Regime 1		Regime 2		Constant	T-Matrix		LL	AIC	SIC
	*λ*_1_	*k*_1_	*λ*_2_	*k*_2_	*β*	*δ*_1_	*δ*_2_			
CHH	− *3.690*E−*08*	− **4.646**	1.450E−07	− **3.361**	**1.033**	**3.240**	− **1.617**	1809	− 3605	− 3574
H	− *4.700*E−*08*	− **4.415**	1.300E−08	− **3.209**	**1.152**	**4.754**	− **3.773**	1624	− 3235	− 3204
HLT	− 7.810E−08	− **3.414**	− 3.710E−08	− **4.521**	**1.055**	**2.110**	− **3.274**	1715	− 3417	− 3386
IHG	− *3.920*E−*08*	− **4.506**	− 2.060E−07	− **3.547**	**1.116**	**5.634**	− **4.766**	1707	− 3401	− 3369
MAR	− 3.550E−08	− **4.396**	1.240E−07	− **3.112**	**1.260**	**4.340**	− **3.054**	1645	− 3275	− 3244
WH	− *4.690*E−*08*	− **4.226**	− 1.140E−06	− **2.773**	**1.227**	**5.314**	− **2.721**	1674	− 3334	− 3303
MGM	− 1.410E−07	− **2.925**	− **6.250E−08**	− **4.198**	**1.540**	**2.953**	− **3.980**	1473	− 2932	− 2900

Note that the bold and italic numbers represent statistical significance and weakly statistical significance at 5$\%$ and 10$\%$, respectively. $\beta$s, representing the sensitivity of the market to hotel stock prices, are statistically significant. $\lambda _2$ for MGM, representing the impact of changes in the number of COVID-19 cases on hotel stock prices in Regime 2, is a statistically significant negative value. $\lambda _1$ for CHH, H, IHG, and WH, representing that in Regime 1, are weakly but statistically significant negative values

**Fig. 15:**
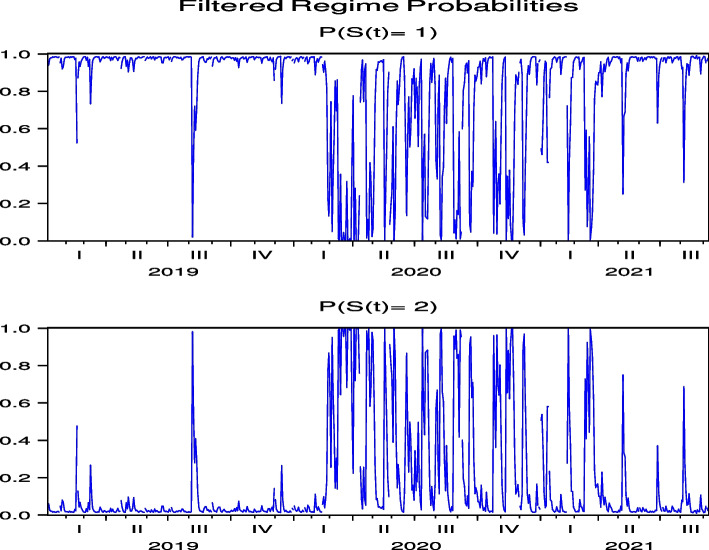
Regime probabilities for CHH. The transition to the high volatility regime continues through after summer of 2021

**Fig. 16:**
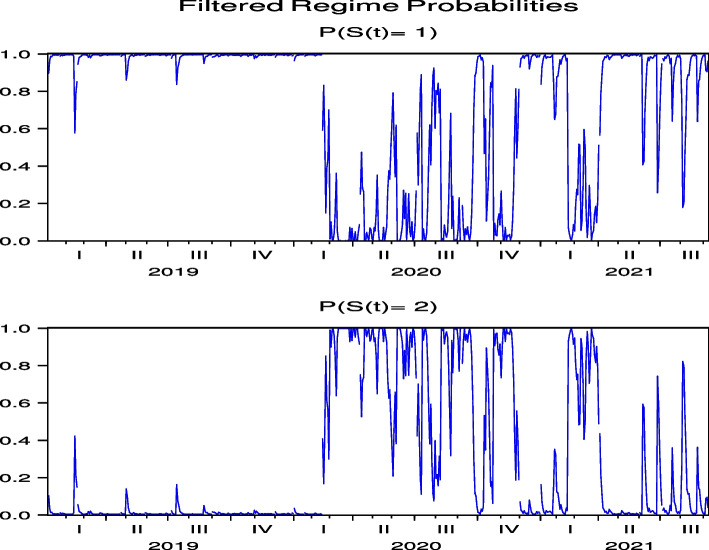
Regime probabilities for H. The transition to the high volatility regime continues through after summer of 2021, similar to CHH

The regime probabilities in Fig. 15 obtained from the hybrid model using the US Hotel stock returns of CHH show that the transition to the high-volatility regime continues until after the summer of 2021, which are more pronounced than those from the direct model in Fig.  7, while the transition to the high-volatility regime continues until after the summer of 2021 in H both by using the direct and hybrid models in Figs. 8 and 16, respectively. Note that Figs. 34, 35, 36, 37 and 38 for HLT, IHG, MAR, WH, and MGM in “Appendix 1” compared with Figs. 24, 25, 26, 27 and 28 for HLT, IHG, MAR, WH, and MGM in “Appendix 1”, respectively, offer the same results of regime probabilities as CHH except for WH. This result demonstrates the COVID-19 impact on US hotel stock prices after eliminating market impact. Based on the analysis using the hybrid regime-switching volatility model, which considers stock market effects, we observe a transition to high volatility in hotel stock prices due to the impact of COVID-19 until around the summer of 2021 in both Japan and the US. Therefore, this result also shows that COVID-19 impacts the price returns of hotel stocks, regardless of whether they are in Japan or the US, if we exclude the impact of the stock market. This result differs from that obtained by using a direct regime-switching volatility model.

Based on these results, because the market affects hotel stock prices simultaneously, COVID-19 indirectly affects Japanese hotel stocks through the Japanese stock market or directly affects them. Meanwhile, US hotel stocks, although negatively affected by COVID-19, have a limited impact from COVID-19 due to the offset of the direct impact of COVID-19 on hotel stocks and no impacts of COVID-19 on the overall stock market. These outcomes are the second contribution of our study, as additional information is obtained from the stock market analyses. Regarding regime probabilities, our analysis of Japanese hotel stock prices using a direct regime-switching volatility model shows that, unlike US hotel stock prices, the regime continued to switch to high volatility from COVID-19 until September 2021. However, in the analysis using the hybrid regime-switching volatility model, which considers stock market effects, we observe a transition to high volatility in hotel stock prices from the impact of COVID-19 until around the summer of 2021 in both Japan and the US. This implies that COVID-19 has an impact on the price returns of hotel stocks, regardless of whether they are in Japan or the US, if we exclude the impact of the stock market. These results are the third contribution of this paper and suggest that the impact of COVID-19 on hotel stock prices may vary from country to country, based on the examples of Japan and the US.

## Robustness checks

### A model robustness check

This study uses a regime-switching model to capture the impacts of regime changes in constant volatility and infection speed on hotel stock price returns. However, it may also be possible to capture price changes through time-varying volatility. This section investigates the time-varying volatility of hotel stock prices and explores the limitations of using the MS-GARCH model as a robustness check. To investigate the heteroskedasticity of hotel stock returns, we conduct ARCH LM tests on the residuals by regressing the log price differences of each hotel’s stock prices on a constant term under the null hypothesis that there is no ARCH effect up to lag=1. From Table 9, except for SB and MGM, we reject the null hypothesis that the squared residuals of both Japanese and US hotel log price differences do not have ARCH effects up to the lag=1 term. Therefore, considering only the preliminary results, we justify the use of the GARCH (1,1) model in modeling the error terms of hotel stock price returns.

**Table 9:** ARCH LM test with null hypothesis of No ARCH with Lag=1

FJT				IMP				RYL			
F-stat.	69.407	P. F(1,586)	0.000	F-stat.	38.530	P. F(1,586)	0.000	F-stat.	42.339	P. F(1,586)	0.000
Obs*R$^2$	62.269	P. Chi$^2$(1)	0.000	Obs*R$^2$	36.276	P. Chi$^2$(1)	0.000	Obs*R$^2$	39.621	P. Chi$^2$(1)	0.000

KYT				OL				SB			
F-stat.	16.228	P. F(1,586)	0.000	F-stat.	16.663	P. F(1,586)	0.000	F-stat.	1.233	P. F(1,586)	0.267
Obs*R$^2$	15.845	P. Chi$^2$(1)	0.000	Obs*R$^2$	16.257	P. Chi$^2$(1)	0.000	Obs*R$^2$	1.235	P. Chi$^2$(1)	0.266

KRT				CHH				H			
F-stat.	192.398	P. F(1,586)	0.000	F-stat.	7.439	P. F(1,627)	0.007	F-stat.	31.408	P. F(1,627)	0.000
Obs*R$^2$	145.337	P. Chi$^2$(1)	0.000	Obs*R$^2$	7.376	P. Chi$^2$(1)	0.007	Obs*R$^2$	30.005	P. Chi$^2$(1)	0.000

HLT				IHG				MAR			
F-stat.	23.846	P. F(1,627)	0.000	F-stat.	17.632	P. F(1,627)	0.000	F-stat.	59.206	P. F(1,627)	0.000
Obs*R$^2$	23.046	P. Chi$^2$(1)	0.000	Obs*R$^2$	17.205	P. Chi$^2$(1)	0.000	Obs*R$^2$	54.270	P. Chi$^2$(1)	0.000

WH				MGM							
F-stat.	64.669	P. F(1,627)	0.000	F-stat.	2.651	P. F(1,627)	0.104				
Obs*R$^2$	58.809	P. Chi$^2$(1)	0.000	Obs*R$^2$	2.648	P. Chi$^2$(1)	0.104				

With the exceptions of SB and MGM, we reject the null hypothesis that the squared residuals from the regression of both Japanese and the US hotel log price differences on a constant do not have ARCH effects up to the lag = 1 term

The modeling and empirical analysis of hotel stock returns described above suggest that COVID-19 might have changed the behavior of price return volatility. From these and the existence of heteroskedasticity, the MS-GARCH model with its multiple regimes appears to be the most effective among those alternatives to capture the process of switching between the high- and low-volatility regimes of hotel stock prices. For simplicity and familiarity, we use the MS-GARCH (1,1) model for this analysis.

6 $$r_t=\epsilon _t,$$

7 $$\epsilon _t=\sigma _t\eta _t,$$

8 $$\sigma _t^2=\left\{ \begin{array}{l} \omega _{1} +\alpha _{1} \epsilon _{t-1}^2+\beta _1 \sigma _{t-1}^2,\\ \omega _{2} +\alpha _{2} \epsilon _{t-1}^2+\beta _2 \sigma _{t-1}^2. \end{array} \right.$$

The transition matrix contains elements $p_{ij}$, which represent the transition probability from state *i* at time $t-1$ to state *j* at time *t*.

9 $$\begin{aligned} P= \begin{pmatrix} p_{11} &{} 1- p_{22} \\ 1-p_{11} &{} p_{22} \end{pmatrix} \end{aligned}$$

Among the estimation results of the MS-GARCH model with two regimes for the seven Japanese hotel stock returns and the NKY return in Table 10, there are no examples in which both regimes satisfy stationarity and of which two regimes show GARCH or ARCH effects, except for RYL. KYT, OL, SB, KRT, and NKY in Table 10 are examples in which both regimes satisfy stationarity and only one regime shows a GARCH or ARCH effect. Therefore, the use of the MS-GARCH model with two regimes is not necessarily robust in this analysis.^15^

**Table 10:** MS-GARCH (1,1) model parameter estimation for Japanese hotel stocks and NKY

	*ω*_1_	*α*_1_	*β*_1_	*ω*_2_	*α*_2_	*β*_2_	*p*_11_	*p*_22_
FJT	**1.285**E−**04**	0.188	2.465E−05	3.495E−04	0.312	0.234	**0.998**	**0.998**
IMP	**1.277**E−**05**	**0.327**	1.000E−06	3.342E−05	(**0.629**)	(**0.705**)	**0.651**	0.085
RYL	**1.743**E−**05**	**0.334**	2.090E−05	**1.345**E−**04**	**0.211**	**0.523**	**0.952**	**0.945**
KYT	2.337E−05	0.347	1.305E−05	2.625E−05	8.661E−02	**0.887**	**0.925**	**0.876**
OL	**1.313**E−**04**	1.261E−06	1.003E−05	7.263E−06	2.564E−02	**0.974**	**0.908**	**0.795**
SB	**1.319**E−**04**	5.544E−02	1.707E−05	**1.623**E−**04**	7.744E−02	**0.695**	**0.998**	**0.998**
KRT	**2.536**E−**04**	1.000E−06	1.983E−05	**3.704**E−**04**	**0.272**	0.388	**0.987**	**0.984**
NKY	7.517E−05	**0.150**	6.684E−05	5.053E−04	0.278	0.000	**0.988**	**0.908**

Note that the bold numbers and round bracketed ones represent statistical significance compared to the corresponding standard errors and no stationarity, respectively. There are no examples except for RYL where both regimes satisfy stationarity and of which two regimes show GARCH or ARCH effects

Of the estimation results of the MS-GARCH (1,1) model with two regimes for the seven US hotel stock returns and SPX returns in Table  11, all results satisfy stationarity with two regimes. Of these, CHH, H, MAR, and WH show GARCH or ARCH effects in both regimes. Thus, it may make sense to use the MS-GARCH model for US hotel stock prices and stock indexes compared to Japanese stock returns. However, one regime for IHG, MGM, and SPX does not have a GARCH or ARCH effect, and the two regimes for HLT remain undetected. Therefore, use of the MS-GARCH model is not necessarily robust in this study. From a qualitative perspective of model selection, because of the non-negativity of the GARCH model, it is not possible to include changes in the number of people infected with COVID-19 in the regimes. Thus, the selected model should have the property that a change in the number of infections does not affect either regime.

**Table 11:** MS-GARCH (1,1) model parameter estimation for US hotel stocks and SPX

	*ω*_1_	*α*_1_	*β*_1_	*ω*_2_	*α*_2_	*β*_2_	*p*_11_	*p*_22_
CHH	**2.502**E−**05**	**0.147**	**0.789**	**5.561E−04**	2.801E−06	**0.789**	**0.973**	**0.840**
H	1.153E−04	**0.129**	1.719E−05	**2.572**E−**05**	**0.136**	**0.839**	**0.997**	**0.993**
HLT	1.753E−04	0.182	3.993E−05	1.856E−03	3.781E−02	1.000E−06	**0.974**	**0.897**
IHG	**1.008**E−**04**	0.224	1.341E−05	1.512E−05	**0.126**	0.859	**0.998**	**0.998**
MAR	**2.083E**−**04**	**0.240**	2.483E−05	9.881E−04	0.245	**0.431**	**0.991**	**0.970**
WH	**1.491E**−**04**	**0.157**	1.194E−05	**5.631**E−**05**	**0.135**	**0.816**	**0.994**	**0.991**
MGM	**2.054**E−**03**	1.005E−06	1.005E−06	5.700E−05	**0.628**	1.005E−06	**0.210**	**0.000**
SPX	**3.684E**−**06**	**0.160**	**0.764**	**3.859**E−**04**	3.057E−05	1.692E−04	**0.970**	**0.704**

Note that the bold numbers represent statistical significance compared to the corresponding standard errors. One regime for IHG, MGM and SPX does not have a GARCH or ARCH effect, and two regimes for HLT remain undetected

### A consistency check with the literature

Anguera-Torrell et al. (2021) found negative impacts of the COVID-19 pandemic on the hotel industry including the US hotels by using a short-term sample period from February 24, 2020 to April 24, 2020. However, the results are completely different from our results from a direct impact model estimation using a long-term sample period, from January 2, 2019 to September 10, 2021. To fill this gap, using the same sample data (February 24, 2020, to April 24, 2020) as in Anguera-Torrell et al. (2021), we obtain the model parameter estimates and regime probabilities for US hotel stock prices.

**Fig. 17:**
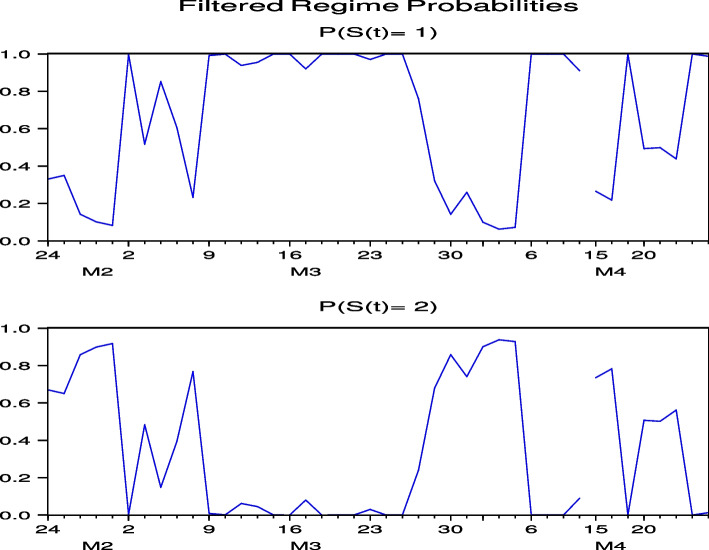
Regime probabilities for CHH. A short-term sample. The regime probabilities were not clearly obtained

**Fig. 18:**
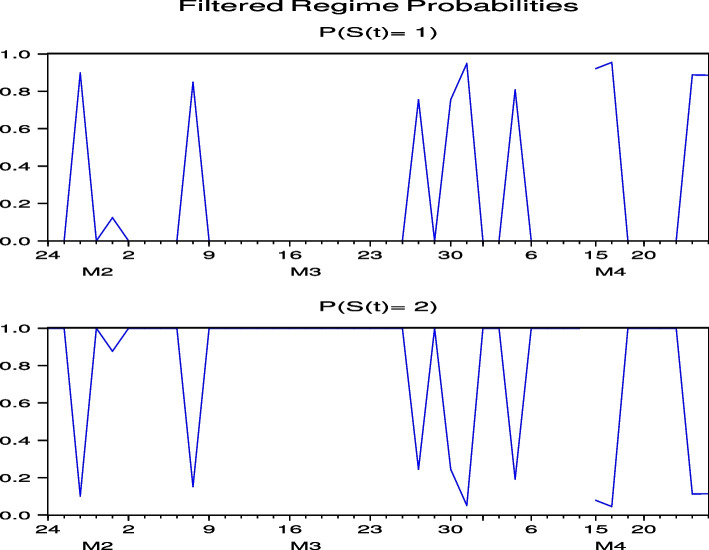
Regime probabilities for H. A short-term sample. The regime probabilities were not clearly obtained, similar to those for CHH

**Fig. 19:**
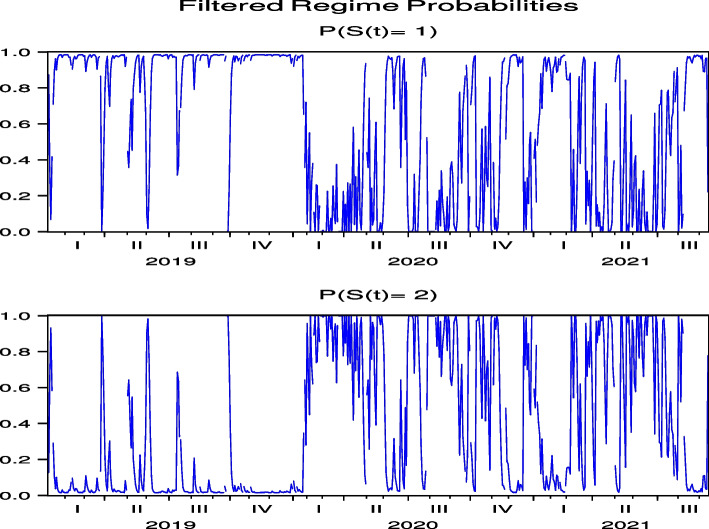
Regime probabilities for RYL. After February 2020, the regime probabilities moved into a regime of high price volatility, then moved into a regime of low price volatility over time

**Fig. 20:**
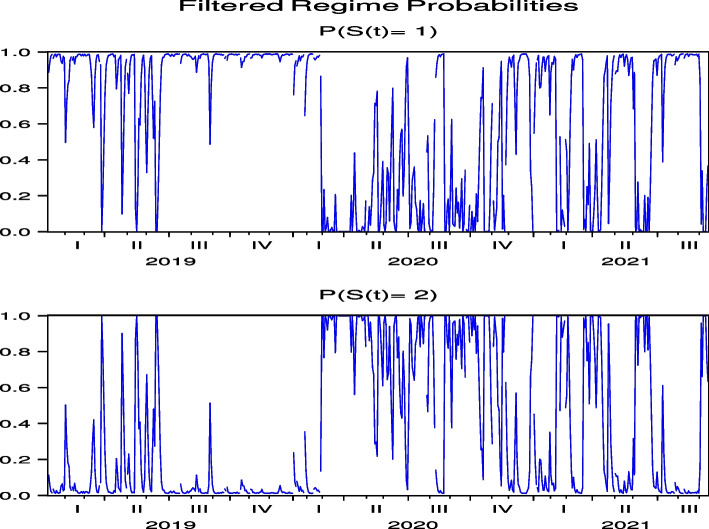
Regime probabilities for KYT. After February 2020, the regime probabilities moved into a regime of high price volatility, then moved into a regime of low price volatility over time

**Fig. 21:**
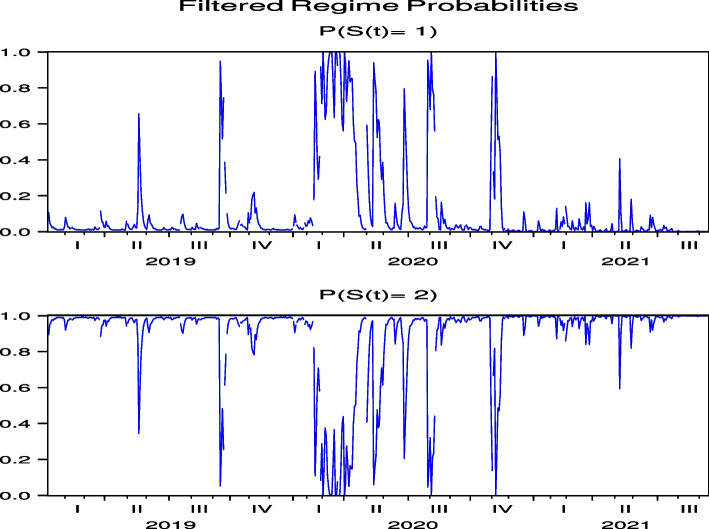
Regime probabilities for OL. After February 2020, the regime probabilities moved into a regime of high price volatility, then moved into a regime of low price volatility soon

**Fig. 22:**
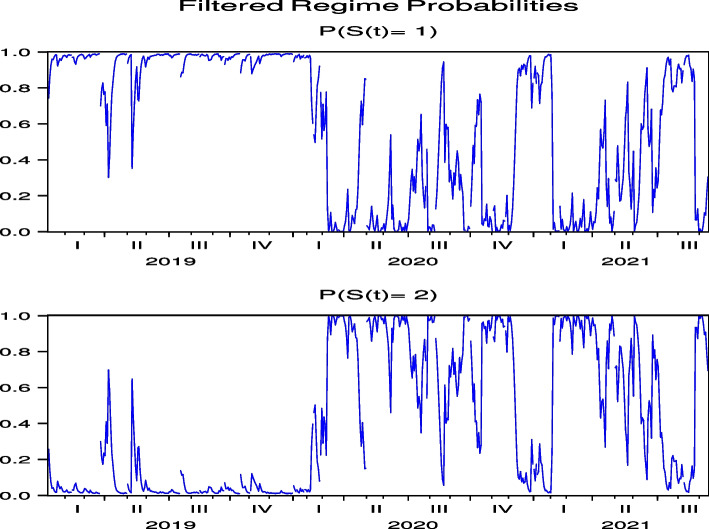
Regime probabilities for SB. After February 2020, the regime probabilities moved into a regime of high price volatility, then moved into a regime of low price volatility over time

**Fig. 23:**
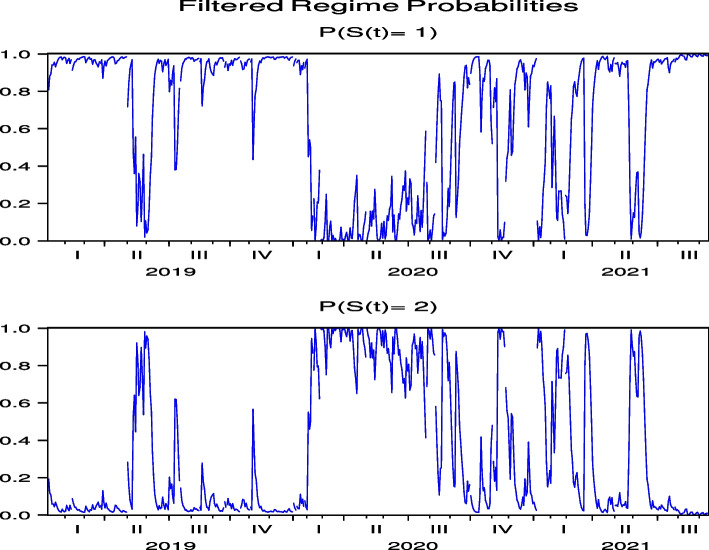
Regime probabilities for KRT. After February 2020, the regime probabilities moved into a regime of high price volatility, then moved into a regime of low price volatility over time

**Fig. 24:**
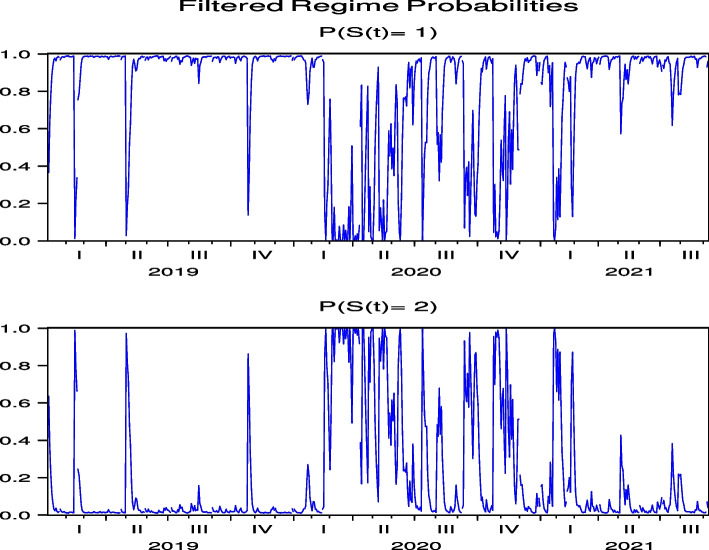
Regime probabilities for HLT. After February 2020, the regime probabilities shifted to a regime with high price volatility, and then converged to a lower volatility regime relatively soon

**Fig. 25:**
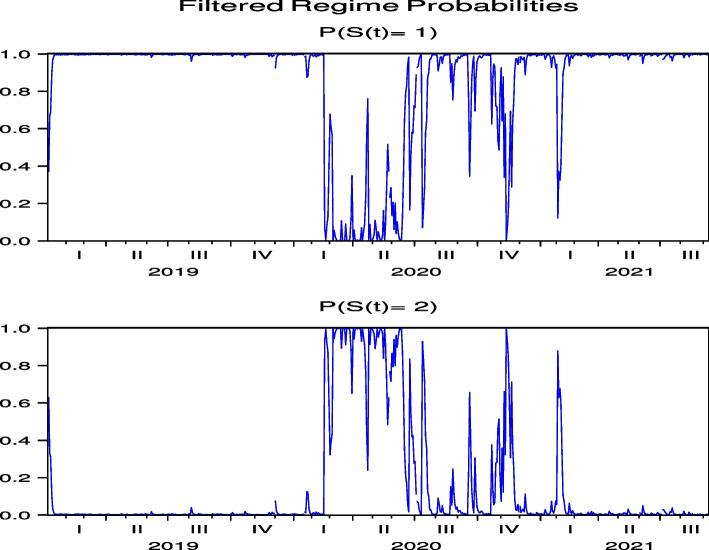
Regime probabilities for IHG. After February 2020, the regime probabilities shifted to a regime with high price volatility, and then converged to a lower volatility regime soon

**Fig. 26:**
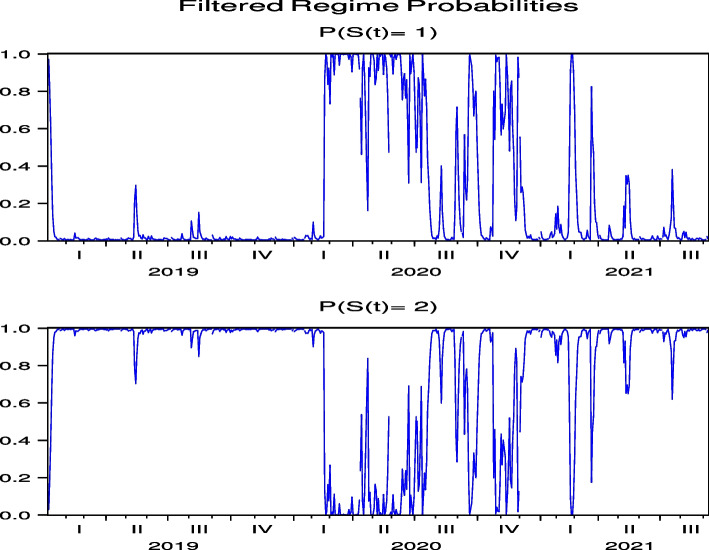
Regime probabilities for MAR. After February 2020, the regime probabilities shifted to a regime with high price volatility, and then converged to a lower volatility regime relatively soon

**Fig. 27:**
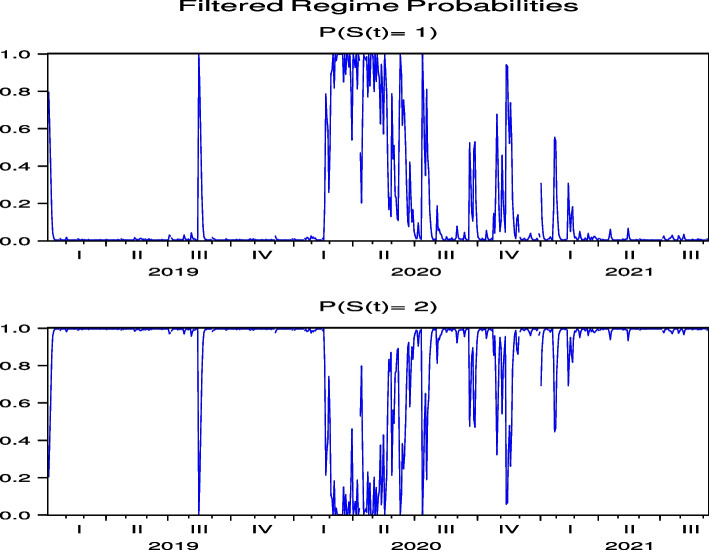
Regime probabilities for WH. After February 2020, the regime probabilities shifted to a regime with high price volatility, and then converged to a lower volatility regime soon

**Fig. 28:**
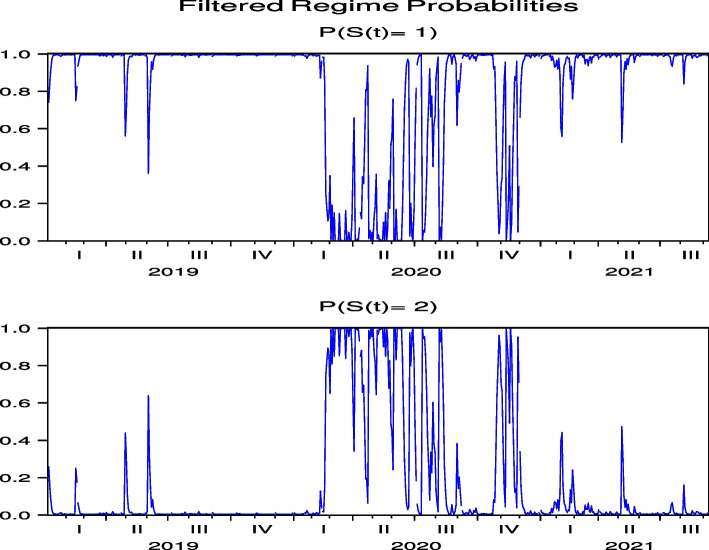
Regime probabilities for MGM. After February 2020, the regime probabilities shifted to a regime with high price volatility, and then converged to a lower volatility regime soon

**Fig. 29:**
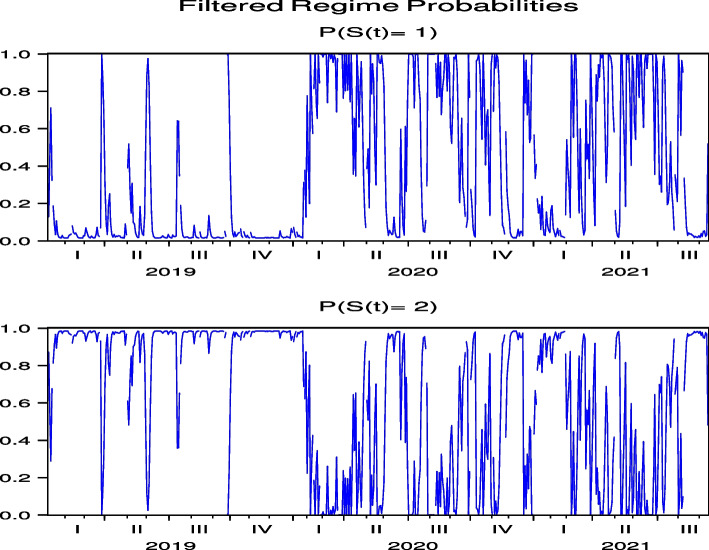
Regime probabilities for RYL. The transition to the high volatility regime continues through after summer of 2021

**Fig. 30:**
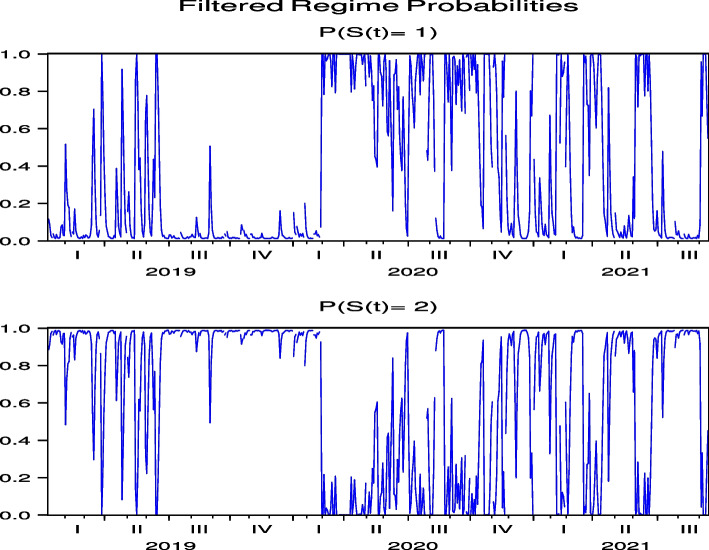
Regime probabilities for KYT. The transition to the high volatility regime continues through after summer of 2021

**Fig. 31:**
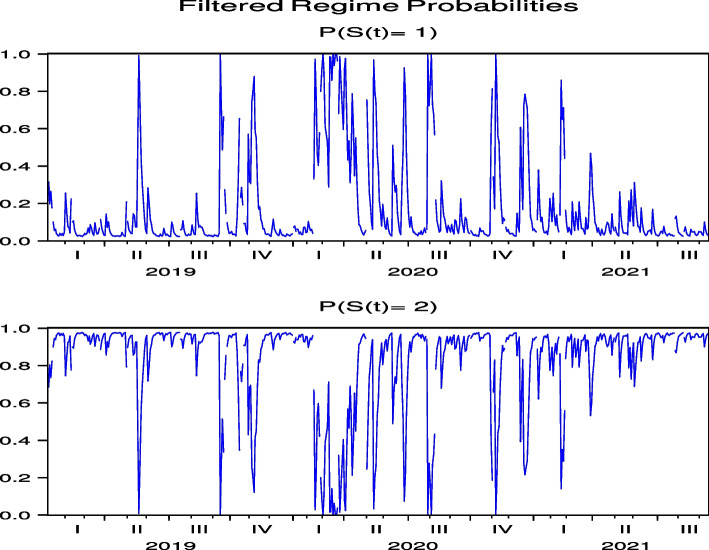
Regime probabilities for OL. The probabilities shifted to a high price volatility regime due to COVID-19 and then converged to a lower one soon

**Fig. 32:**
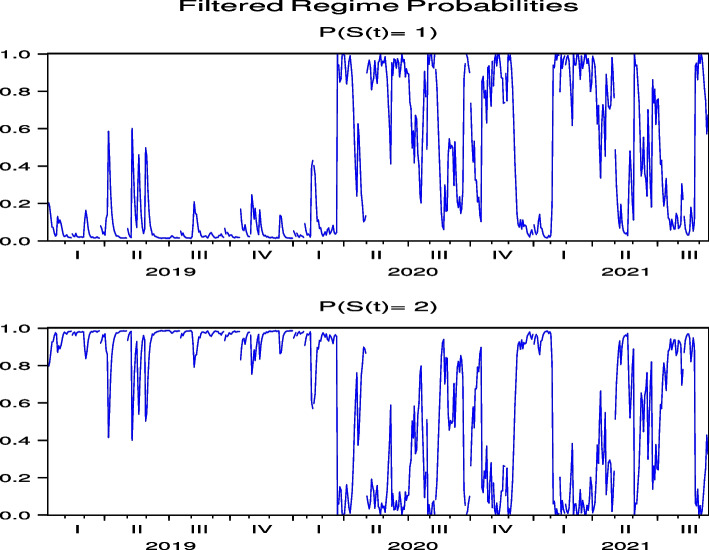
Regime probabilities for SB. The transition to the high volatility regime continues through after summer of 2021

**Fig. 33:**
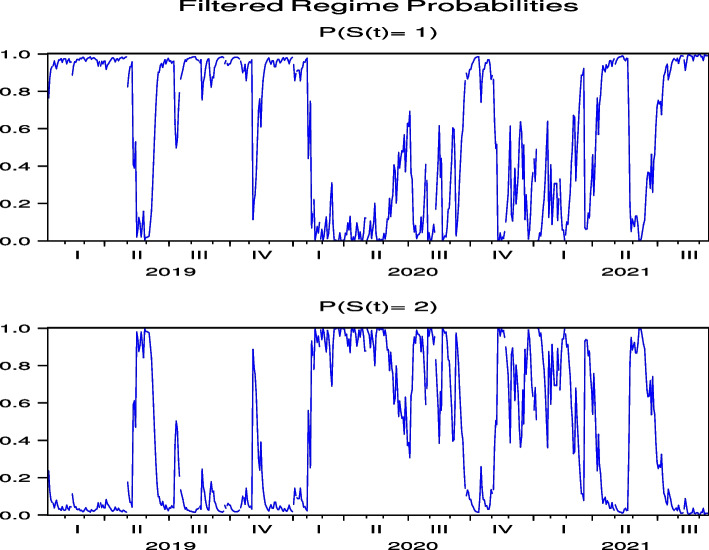
Regime probabilities for KRT. The transition to the high volatility regime continues through almost after summer of 2021

**Fig. 34:**
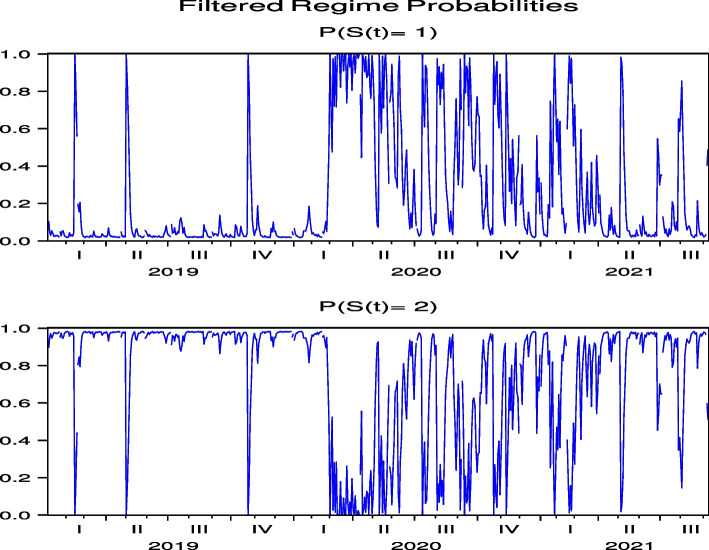
Regime probabilities for HLT. The transition to the high volatility regime continues through after summer of 2021

**Fig. 35:**
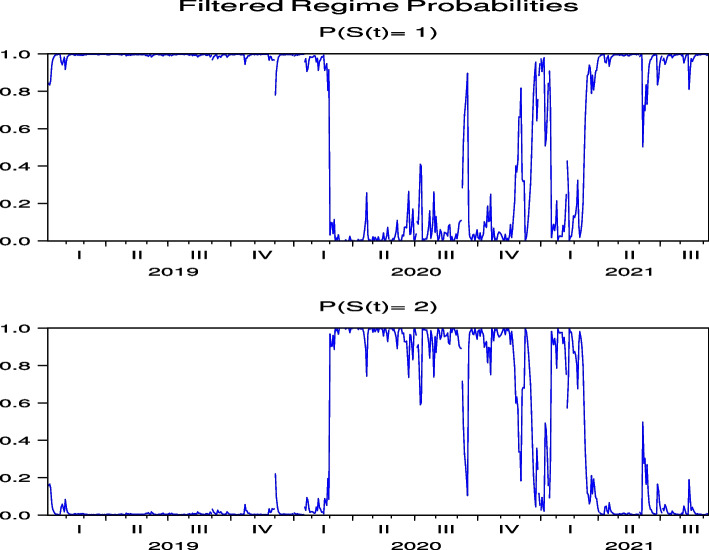
Regime probabilities for IHG. The transition to the high volatility regime continues through almost after summer of 2021

**Fig. 36:**
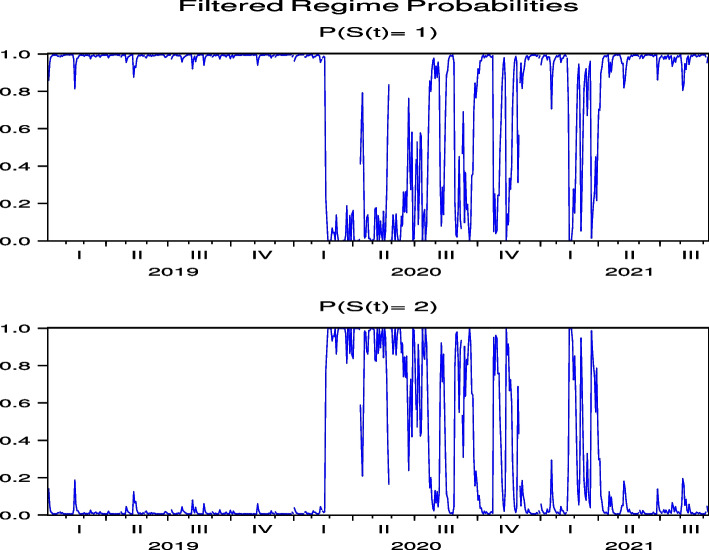
Regime probabilities for MAR. The transition to the high volatility regime continues through almost after summer of 2021

**Fig. 37:**
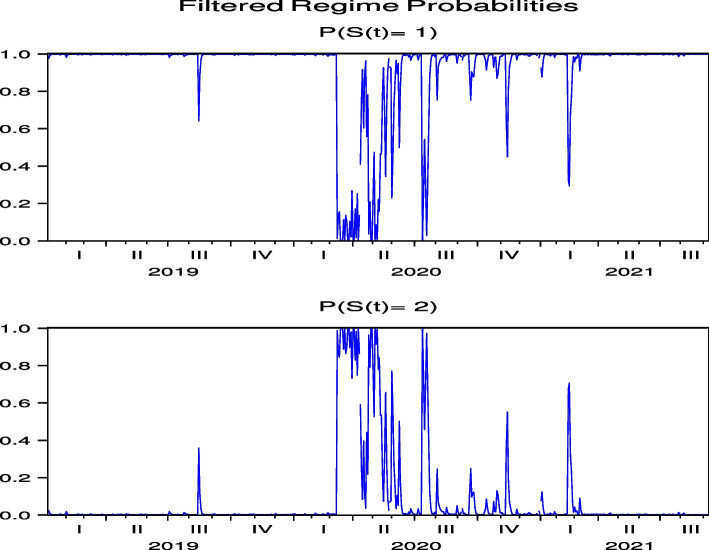
Regime probabilities for WH. The transition to the high volatility regime stops through after summer of 2021

**Fig. 38:**
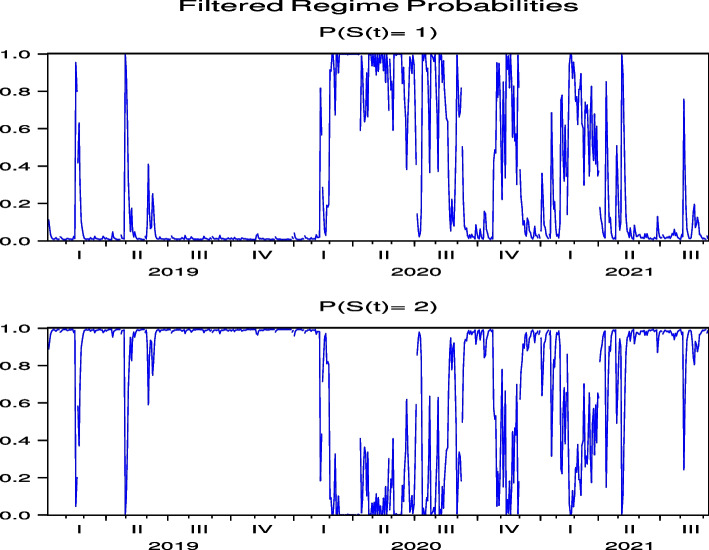
Regime probabilities for MGM. The transition to the high volatility regime continues through almost after summer of 2021

**Fig. 39:**
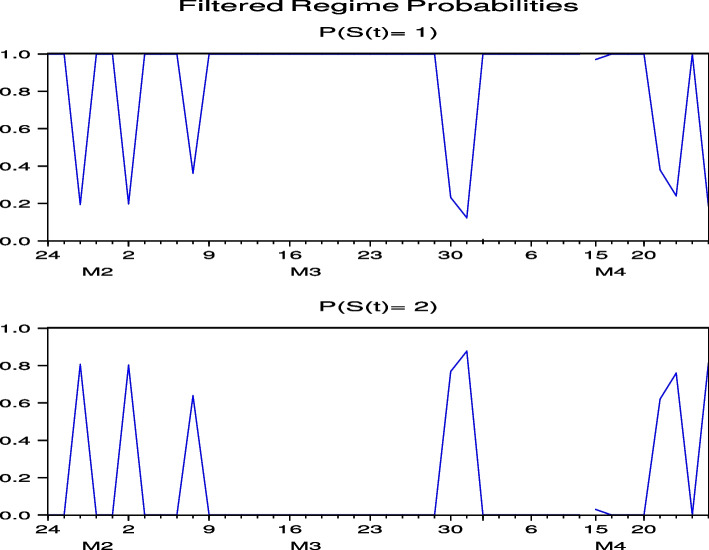
Regime probabilities for HLT. A short-term sample. The regime probabilities were not clearly obtained, similar to those for CHH and H

**Fig. 40:**
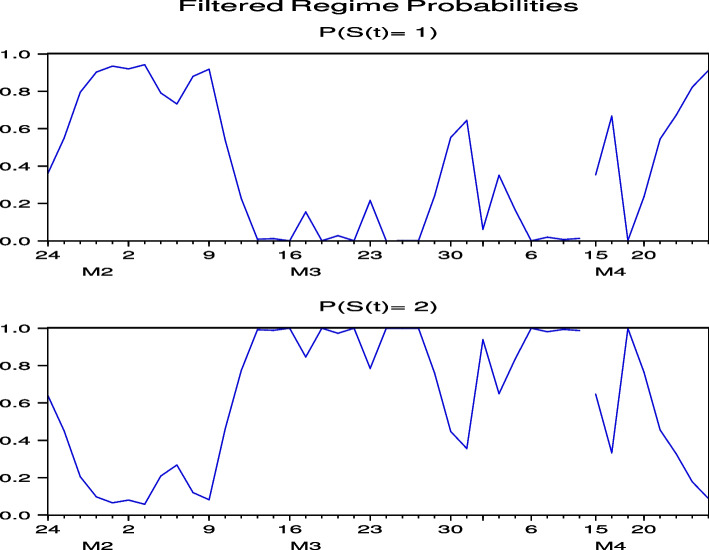
Regime probabilities for IHG. A short-term sample. The regime probabilities were not clearly obtained, similar to those for CHH and H

**Fig. 41:**
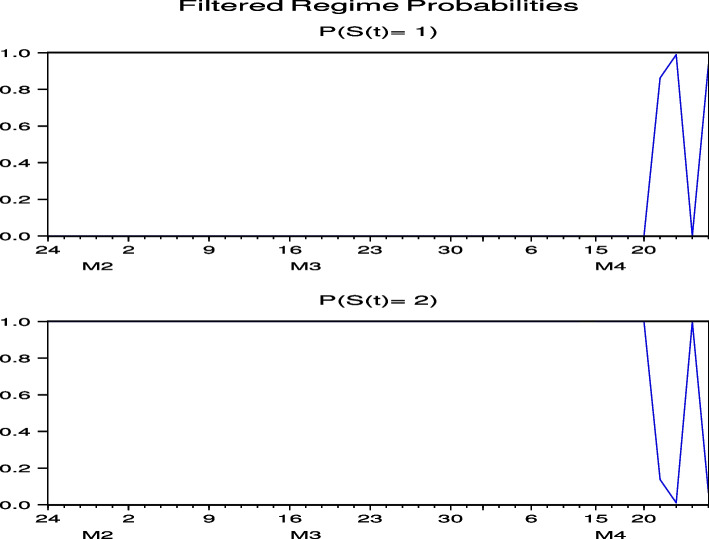
Regime probabilities for MAR. A short-term sample. The regime probabilities were not clearly obtained, similar to those for CHH and H

**Fig. 42:**
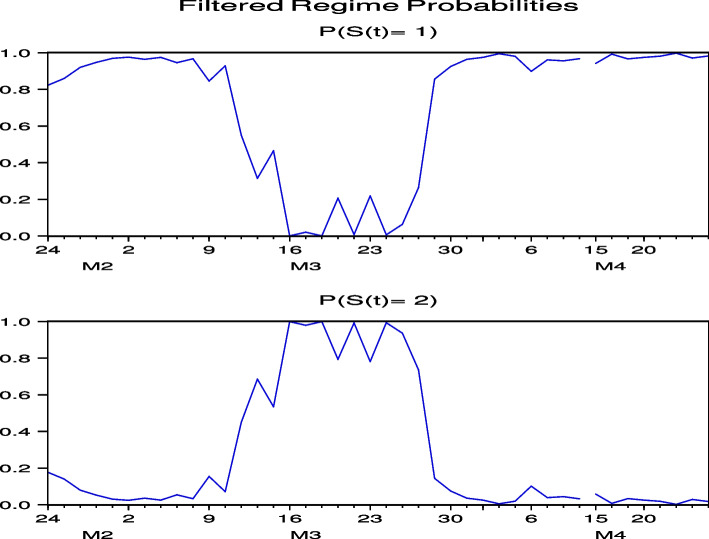
Regime probabilities for WH. A short-term sample. The figure exhibits relatively clear regime-switching

**Fig. 43:**
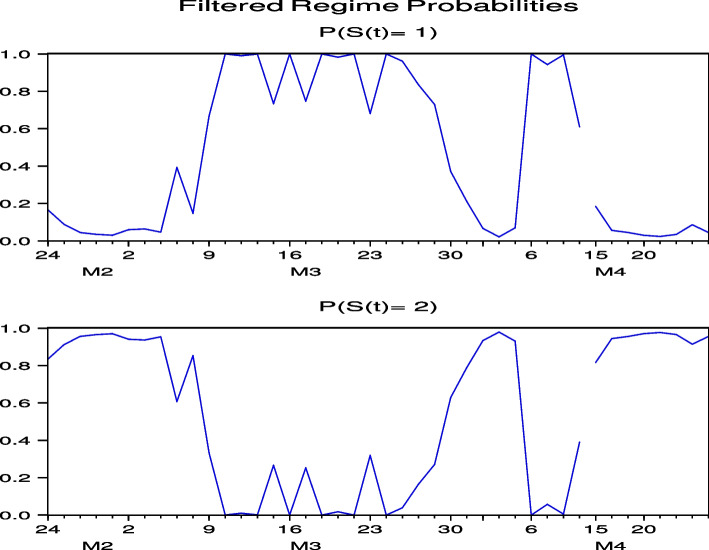
Regime probabilities for MGM. A short-term sample. The figure exhibits relatively clear regime-switching, similar to WH

Table 12 presents the total estimation results for CHH, H, HLT, IHG, MAR, WH, and MGM. Using a short period of data, we obtain statistically significant and negative $\lambda _1$ or $\lambda _2$ for CHH, H, and MAR. The results indicate that the infection speed of COVID-19 affects the short-term analysis more, focusing only on the first wave of COVID-19, as in the case of Japanese hotel stocks. This differs from our results without the impact of the speed of infection on US hotel price returns using the direct model for a longer-term sample from January 2, 2019, to September 10, 2021. However, this is consistent with the results of Anguera-Torrell et al. (2021). Thus, the difference between our analysis, using the direct model for the long-term sample, and that of Anguera-Torrell et al. (2021) comes from the difference in the selection of the sample period. By contrast, there are a number of cases in which the transition probabilities of $\delta _{1}$ or $\delta _{2}$ were not statistically significant, except for WH and MGM, resulting in the regime probabilities shown in Figs. 17 and 18 for CHH and H, respectively, where we did not clearly obtain the regime probabilities. Note that Figs. 39, 40 and 41 for HLT, IHG, and MAR, respectively in “Appendix 1” offer the same regime probability results for CHH and H, while Figs. 42 and 43 for WH and MGM in “Appendix 1”, respectively, exhibit relatively clear regime-switching. These results indicate that the short-term analysis focusing only on the first wave of COVID-19 does not capture the regime-switch volatility well. These results differ from our analysis using a longer-term sample from January 2, 2019, to September 10, 2021, in which we can capture the existence of regime-switching volatility. Thus, we show that the analysis using short-term data from February 24, 2020 to April 24, 2020, only before and after the first wave of COVID-19, has the disadvantage of not accurately capturing the volatility regime-switching and the more pronounced impact of infection speed on price returns, implying the importance of selecting a sufficient sample size to capture the impact of COVID-19 on hotel stock prices. “Appendix  2” presents the results for the Japanese hotel stocks.

**Table 12:** Direct model parameter estimation for US hotel stocks: a short-term sample immediately before and after COVID-19

	Regime 1			Regime 2			T-Matrix	
	*C*_1_	*λ*_1_	*k*_1_	*C*_2_	*λ*_2_	*k*_2_	*δ*_1_	*δ*_2_
CHH	4.680E−04	1.070E−06	− **2.604**	− **2.577E−02**	− **4.390E−06**	− **4.248**	**1.784**	− 1.038
H	− **8.138E−03**	− **1.470E−06**	− **6.311**	− 1.326E−02	− 2.090E−06	− **2.536**	− 0.644	− **1.529**
HLT	− 1.025E−02	− 8.480E−07	− **2.801**	− **8.845E−03**	3.970E−07	− **6.272**	**1.872**	1.221
IHG	− **1.929E−02**	− 1.000E−06	− **3.658**	− 8.612E−03	4.250E−06	− **2.422**	2.178	− **2.468**
MAR	− **8.404E−03**	− **1.420E−05**	− **9.545**	− 1.187E−02	1.510E−07	− **2.623**	− 9.147	− **2.198**
WH	5.030E−04	− 5.140E−06	− **2.949**	− 6.516E−02	3.940E−05	− **2.156**	**3.424**	− **2.141**
MGM	− 3.457E−02	3.080E−05	− **1.848**	− **2.903E−02**	2.670E−06	− **3.282**	**1.913**	− **2.385**

Note that the bold numbers represent statistical significance at 5$\%$. We obtain statistically significant and negative $\lambda _1$ or $\lambda _2$, representing the impact of changes in the number of COVID-19 cases on hotel stock prices in Regime 1 or 2, respectively, for CHH, H and MAR. In addition, there were a number of cases where the transition probabilities of $\delta _{1}$ or $\delta _{2}$ were not statistically significant except for WH and MGM

## Discussion of findings

### A comparative analysis with previous studies

Research on the impact of COVID-19 on the financial and commodity markets is accelerating. Zaremba et al. (2020) demonstrated that government interventions significantly and robustly increase the volatility in international stock markets as the first attempt to examine the influence of non-pharmaceutical policy responses to the COVID-19 pandemic. Zhang et al. (2020) empirically showed that global financial market risks have increased substantially in response to the pandemic by using the data up to March 27, 2020. By examining the safe haven property of gold for the crude oil market during this phase, Dutta et al. (2020) showed that gold outperforms Bitcoin as a safe haven asset. Akhtaruzzaman et al. (2021) found that financial contagion occurs through increased dynamic conditional correlations during the COVID-19 period. Using daily COVID-19 confirmed cases and deaths and stock market return data from 64 countries over the period January 22, 2020, to April 17, 2020, Ashraf (2020) found that stock markets responded negatively to the growth in COVID-19 confirmed cases. By analyzing the time-frequency relationship between the COVID-19 outbreak, oil price, geopolitical risk, economic uncertainty, and the US stock market, Sharif et al. (2020) showed that the COVID-19 outbreak has a greater effect on US geopolitical risk and economic uncertainty than on the US stock market, and oil is leading the US market at low and high frequencies. Using daily data from Canada and the US, Xu (2021) found there is a negative effect of an increase in COVID-19 cases on the stock market in general. Baig et al. (2021) showed that increase in COVID-19 cases/deaths increase stock market volatility and illiquidity. As an indirect impact model of COVID-19 based on a regime-switching model in which the number of infected people influences regime changes, Kanamura (2021) empirically examined the impact of COVID-19 on the price volatility of various asset classes by focusing on timing. Athari et al. (2022) examined the impact of the world pandemic uncertainty index on the German stock market index (DAX index) for the period from the first quarter of 1996 to the third quarter of 2020 by using Markov regime-switching and Fourier based approaches. Athari and Hung (2022) explored the time–frequency return connectedness of the four most relevant asset classes of equity, digital assets, commodity, and fixed income by employing the wavelet analysis for daily data over the period from February 2017 to September 2021. These studies, in particular, those using information on COVID-19 explicitly or implicitly, are quite meaningful for examining the impact of COVID-19 on stock and commodity markets comprehensively, but they do not provide a specific analysis of the travel and tourism industry, particularly the hotel industry, which we aim to examine.

Studies have examined the impact of exogenous risks on travel and tourism stocks. Demiralay and Kilincarslan (2019) analyzed the vulnerability of four regional travel and leisure industry stock indexes to geopolitical risks. García-Gómez et al. (2021) showed that there is a negative influence of the outbreaks of the diseases on stock returns of hotels in the US and the impact of COVID-19 is incomparably higher in magnitude compared to previous diseases. Yiwei et al. (2021) investigated the spillover effect of industrial sectors by emphasizing the tourism sector in China and the US between 2019 and 2020 (pandemic period) using the multivariate generalized autoregressive conditional heteroskedastic-dynamic conditional correlation (MGARCH-DCC) and continuous wavelet transform (CWT) techniques. Carter et al. (2021) investigated the stock market performance from the second half of February through the latter portion of March 2020 for the US travel-related firms (airlines, restaurants, and hotels) in response to the pandemic. Irani et al. (2022) investigated the impacts of country-specific risk, namely, political and economic risks, global economic policy uncertainty, and also macroeconomic factors on the price of Turkish tourism firms’ stocks during the 2000 to 2017 period. However, these studies dealt with the early stages of COVID-19 except for pre-COVID-19 analyses of Demiralay and Kilincarslan (2019) and Irani et al. (2022), and they did not directly address information on COVID-19-infected patients.

Subsequently, many researchers have examined the performance of hotel stocks during COVID-19 by directly dealing with COVID-19 information, such as the number of people infected with COVID-19. Kaczmarek et al. (2021) addressed the characteristics that may protect stock market-listed companies from the tourism sector against the COVID-19 pandemic by assessing the relationships between the company and country characteristics and their stocks’ reaction to COVID-19 based on the machine learning tool Elastic net and Fama–MacBeth regressions. Interestingly, the results of Kaczmarek et al. (2021) indicated that countries with less individualism may be better positioned to cope with a pandemic. Anguera-Torrell et al. (2021) estimated how the 20 world-largest and publicly listed hotel companies’ stock market returns reacted to the pandemic evolution and the different public sector economic measures across the different countries from February 24, 2020 to April 24, 2020 by using regression techniques and the room number weighted average of the growth rate of COVID-19 diagnosed cases. Using regression techniques, they showed that the hotel industry has been negatively affected by the COVID-19 evolution and that hotel stock prices are positively correlated with economic policies, with a direct impact on the public budget. Wu et al. (2021) explored the effects of the COVID-19 outbreak on stock price movements of China’s tourism industry by using an event study method, resulting in the negative impacts of the crisis on tourism sector stocks’ abnormal returns based on the daily growth rate of COVID-19 confirmed cases, that is, the speed of increase. Chen et al. (2020) analyzed the impact of COVID-19 government restrictions on US travel and leisure companies, resulting in no effect of the growth of COVID-19 confirmed cases on stock returns. These studies explicitly use COVID-19 information, including infection speed, as the impact of COVID-19 but do not model the regime changes in volatility that characterize the impact of COVID-19.

By contrast, Lin and Falk (2021) investigated the performance of the stock market and its volatility in the travel and leisure industry for three Nordic countries using daily data from June 2018 to June 2020, a period that includes the first wave of COVID-19 pandemic, based on the Markov regime-switching model. Baek et al. (2020) used the MS-AR (1) model to confirm a regime change in the US stock market volatility with the inception of COVID-19 and show a significant increase in total risk for the US stock market, including restaurants, hotels, and lodging industry by using the information of COVID-19. However, these studies do not use a regime-switching model in which returns are directly affected by COVID-19.

Thus, to the best of our knowledge, there appear to be gaps in the existing research that are yet to be filled. There is no analysis, to the best of our knowledge, that examines the direct impact of COVID-19 on price returns and regime-switching volatilities simultaneously; or investigates the impact of COVID-19 on the stock prices of the Japanese hotel industry. This study focuses on Japanese hotel stocks and compares them with US stocks, and analyzes the impact of COVID-19 on these stocks using two new regime-switching volatility models whose returns are affected by the spread of the infection with and without stock market impacts.

The parameter estimation of the former model found that the infection speed negatively affects Japanese hotel stock returns, unlike in the US. The latter model, which can remove the impact of the stock market on regime-switching volatility, demonstrates that COVID-19 negatively affects hotel stock prices, regardless of whether they are in Japan or the US. Additionally, our analysis of Japanese hotel stock prices using the former model showed that, unlike US hotel stock prices, the regime continued to switch to high volatility during COVID-19 until September 2021. However, using the latter, whose regime-switching excludes the impact of the stock market, we observe a transition to high volatility in hotel stock prices due to the impact of COVID-19 until around summer 2021 in both Japan and the US. These results suggest that COVID-19 is likely to have an impact on hotel stock prices in general, except for the influence of the stock market, and considering the market influence, COVID-19 directly and/or indirectly affects Japanese hotel stocks through the Japanese stock market; however, US hotel stocks have limited impacts from COVID-19 due to the offset between the influence on hotel stocks and the no-effect on the stock market. The results for US hotels are consistent with those of Chen et al. (2020).

### An analysis of health and economic policies on COVID-19

Because the sample period includes both the initial reaction to COVID-19 and subsequent policy responses, it is crucial to account for policies including both health and economics in each of the respective countries during the sample period. This subsection presents an analysis of the health and economic policies related to COVID-19. In terms of health policy, the United States was one of the earliest countries to initiate vaccination on December 14, 2020. The Biden administration surpassed 100 million vaccinations in just 59 days since its inauguration in January 2021 and 200 million vaccinations in its 92nd day in office, far exceeding the initial forecasts. By contrast, in Japan, the vaccination of healthcare workers against the new coronavirus finally started at medical institutions nationwide on February 17, 2021, two months later than in the US. As of April 1, 2021, the rate per 100 people in Japan was only 0.84 compared with 52.49 in the US, according to the website of Our World in Data. As of August 1, 2021, the rate per 100 population reached 80.93 in Japan compared with 105.62 in the US, according to the website. Thus, it can be seen that the US has responded more quickly to the health policy on COVID-19 than Japan.

Meanwhile, looking at the economy, the US implemented a lockdown but removed restrictions in many places on the economy in July 2021 and steered the economy towards an early recovery (USA 2021), whereas Japan did not implement a lockdown but imposed moderate restrictions, which resulted in a slower economic recovery.

As a result, the real GDP growth in 2021 was 5.7$\%$ in the US, compared to 1.6$\%$ in Japan, according to the IMF website. The differences in health and economic policies between the US, which responded quickly and robustly to the COVID-19 crisis, and Japan, which responded slowly and with an eye on the situation in surrounding countries, may have contributed to the differences in our results between the US, where the impact of COVID-19 on hotel stock prices was relatively short-lived, and Japan, where the impact was more prolonged.

### Practical and political implications

The results obtained in this study argue that COVID-19 is likely to have an impact on hotel stock prices in general, except for the influence of the stock market, and considering the market influence, COVID-19 directly and/or indirectly affects Japanese hotel stocks through the Japanese stock market; however, US hotel stocks have limited impacts from COVID-19 owing to the offset between the influence on hotel stocks and the no-effect on the stock market. The implication for investors and portfolio managers from this study is that, for hotel stock investments, the impact of COVID-19 on hotel stock returns depends on the balance between direct effects, such as the number of people infected, and indirect effects, such as the market, and varies by country and region. In particular, investors in Japanese hotel stocks should be aware that they may be vulnerable to the direct or indirect effects of crises, such as COVID-19, either directly or indirectly through the market.

The implications of this study for tourism management, albeit a comparison between Japan and the US, suggest that the impact of COVID-19 on hotel company value varies from country to country if we consider hotel stock prices to embody hotel companies or project value. Tourism management officials should be able to predict the impact of COVID-19 on hotel projects in the relevant country by examining in detail the impact of COVID-19 on the stock price in each country. The implication for monetary policymakers is that, in response to economic crises such as COVID-19, market surveillance authorities need to monitor the impact of the crisis on the stock market, that is, whether the stock market is an amplifier that magnifies the impact of the crisis, a buffer that mitigates the impact of the crisis, or neither. This monitoring would provide proactive warnings to market participants about the effects of the crisis, and thus increase the likelihood of preventing a market crash.

### Policy recommendations

Given the magnitude of the stock market’s impact on hotel stock prices obtained in this study, our monetary policy recommendation is to strengthen stock market risk management. With this strengthening, we believe that individual company stocks such as hotels will trade healthier on the stock market.

Since hotel stock prices were affected by the number of new COVID-19 cases in this study, accurate knowledge of the number of new COVID-19 cases is valuable not only in the medical field but also in the business field, such as hotel stock trading. Therefore, a recommendation for healthcare policies is to establish a system that accurately identifies the number of new COVID-19 cases.

Although this study is merely an analysis of the differences between Japan and the US, we found that the impact of COVID-19 on the business-related quantitative value, namely, hotel stock prices, varies greatly from country to country. In particular, while the US recovered from the effects of COVID-19 in a relatively short period, the effects of COVID-19 persisted in Japan for a relatively medium to long period. Neither of these is necessarily good but may reflect the national character of the people who primarily trade in them. In this light, the recommendation for government policy is to develop a comprehensive government policy that takes into account all aspects of the economy and healthcare, in line with the national character that is highlighted through the results of economic analyses, such as this study.

### Financial flexibility and COVID-19

By comparing the impact of COVID-19 on Japanese hotel stocks with an analysis of the US hotel stocks, we have empirically shown, unlike the US hotel stock prices, that Japanese hotel stock returns are negatively affected by the speed of COVID-19 infection and that the impact of the high-volatility regime continues until September 2021. However, not all Japanese and US hotel stocks show these characteristics. Specifically, the price returns of the Japanese IMP were not negatively affected by the COVID-19 infection speed in Table 4. In addition, those of H in the US have been affected by COVID-19 in the high-volatility regime for a relatively long time, as shown in Fig. 8, although not as explicitly as Japanese hotel stocks. By delving deeper into the reasons for these exceptions, we explore the factors affecting hotel stock prices. Liu et al. (2021) observed that the positive effect of operating flexibility on cumulative abnormal returns exists only for firms with light assets measured by the fixed asset ratio (net fixed assets scaled by total assets). Thus, our findings may be affected by fixed asset ratios. According to Table 13, the property, plant, and equipment (PPE) ratios of Japanese hotels are generally higher than those of US hotels. Therefore, Japanese hotels are less flexible in terms of management and more susceptible to COVID-19. This may have contributed to the continued shift towards high long-term volatility in Japanese hotel stocks. This is consistent with the results of IMP, a Japanese hotel with a low PPE ratio of 25$\%$ in Table 13, compared with the other Japanese hotels with 48$\%$ to 85$\%$, and unaffected by the speed of COVID-19 infection in Table 4, and those of H, a US hotel with a high PPE ratio of 39$\%$, compared to the other US hotels with 6$\%$ to 23$\%$, except for MGM, and continuing high regime probability in Fig. 8. Therefore, the asset-first structure of the Japanese hotel industry may contribute to the long-term impact of COVID-19 on hotel stock prices. Conversely, there are an important indication that Japanese hotel stocks may be able to mitigate the impact of COVID-19 if a light-asset strategy is adopted, even for Japanese hotels.

**Table 13:** PPE ratios during COVID-19 period

As of 3/30/2021	FJT	IMP	KYT	RYL	OL	SB	KRT
Total assets	96,595,000	65,420,000	17,084,932	61,867,000	1,040,465,000	1,698,497,000	239,032,000
Net PPE	58,471,000	16,412,000	14,433,815	42,159,000	665,557,000	1,445,042,000	114,907,000
Net PPE/total assets $\%$	61	25	84	68	64	85	48
(JPY in thousands)							

As of 12/30/2020	CHH	H	HLT	IHG	MAR	WH	MGM
Total assets	1,587,333	9,129,000	16,755,000	5,039,000	24,701,000	4,644,000	36,494,930
Net PPE	372,892	3,600,000	1,118,000	504,000	2,266,000	278,000	22,918,780
Net PPE/total assets $\%$	23	39	7	10	9	6	63
(USD in thousands)							

Data is obtained from Investing.com. IMP, a Japanese hotel, has a low PPE ratio of 25$\%$, compared to the other Japanese hotels with 48$\%$ to 85$\%$. H, a US hotel, has a high PPE ratio of 39$\%$, compared to the other US hotels with 6$\%$ to 23$\%$ except for MGM

## Conclusions

This study empirically examined the impact of COVID-19 on asset price returns in the Japanese hotel industry relative to the US, considering the role of stock markets. This study makes three main contributions to the literature. First, it proposed two new regime-switching volatility models based on econometric analysis, in which the speed of COVID-19 infection directly affects price returns, and both COVID-19 and the stock market affect these returns. Second, using relatively long-term data from January 2019 to September 2021, we found that Japanese hotel stock returns are negatively impacted by the infection speed in a direct or indirect way via the corresponding market, while US hotel stock returns have a limited impact from COVID-19 owing to offsets between the direct impact of COVID-19 on hotel stocks and no impact of COVID-19 on the overall stock market. Third, and most importantly, our analysis of Japanese hotel stock prices using a direct regime-switching volatility model showed that, unlike US hotel stock prices, the regime continued to switch to high volatility from COVID-19 until September 2021. However, the analysis using the hybrid regime-switching volatility model, whose regime-switching excludes the impact of the stock market, revealed a transition to high volatility in hotel stock prices due to the impact of COVID-19 until around the summer of 2021 in both Japan and the US. The results of this study imply that investors and portfolio managers should be aware that in hotel stock investments, the impact of COVID-19 on hotel stock returns depends on the balance between direct effects, such as the number of people infected, and indirect effects, such as the market, which varies from country to country and region to region.

In this analysis, we used US hotel stocks, which undertake business internationally, for comparison with the Japanese hotel stocks we are interested in, but an empirical analysis of European and Asian hotel stocks is also possible. We were unable to include changes in the number of new COVID-19 infections in the MS-GARCH (1,1) model because of the non-negativity of the GARCH process. We believe that this is another limitation of this study. Furthermore, the results of the empirical analysis of Japanese hotel stocks may differ depending on the future behavior of the number of infected people. These analyses will be the subject of future research, depending on the availability and accumulation of data.

## Figures and Tables

**Fig. 44:**
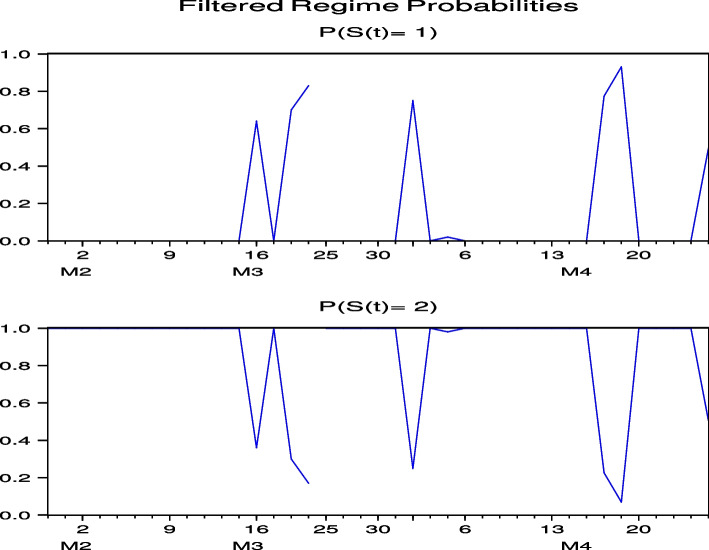
Regime probabilities for FJT. A short-term sample

**Fig. 45:**
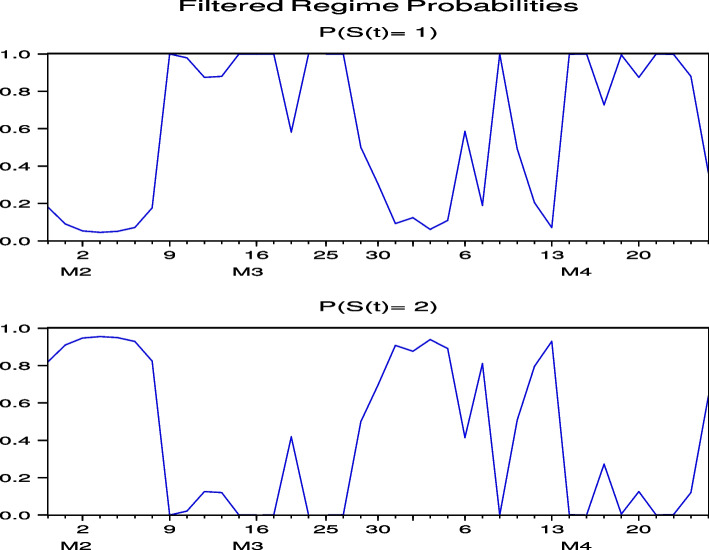
Regime probabilities for IMP. A short-term sample

**Fig. 46:**
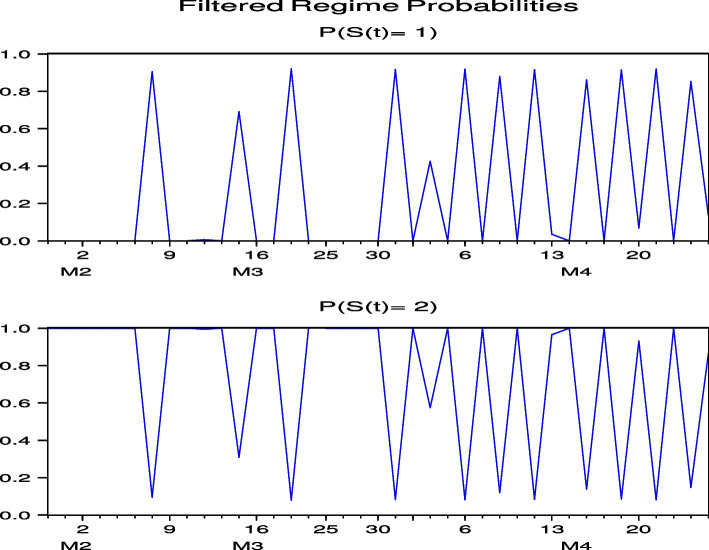
Regime probabilities for RYL. A short-term sample

**Fig. 47:**
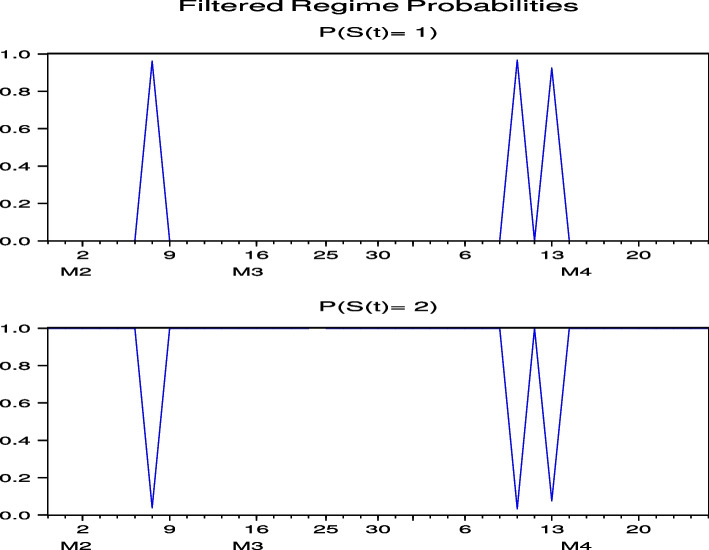
Regime probabilities for KYT. A short-term sample

**Fig. 48:**
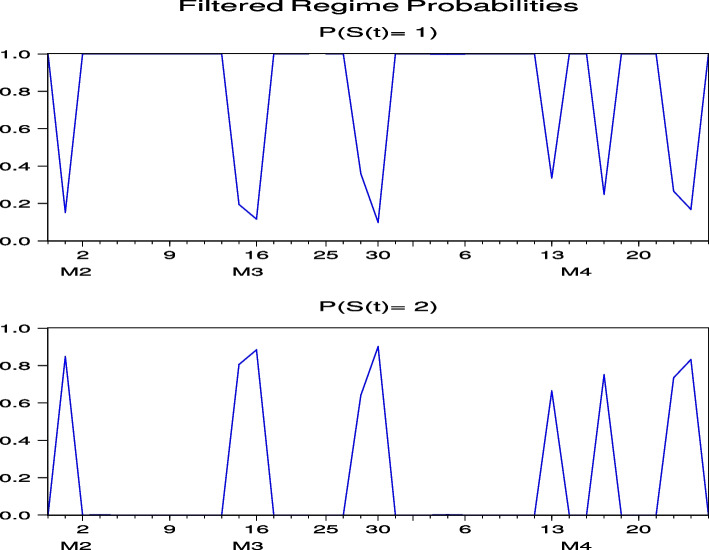
Regime probabilities for OL. A short-term sample

**Fig. 49:**
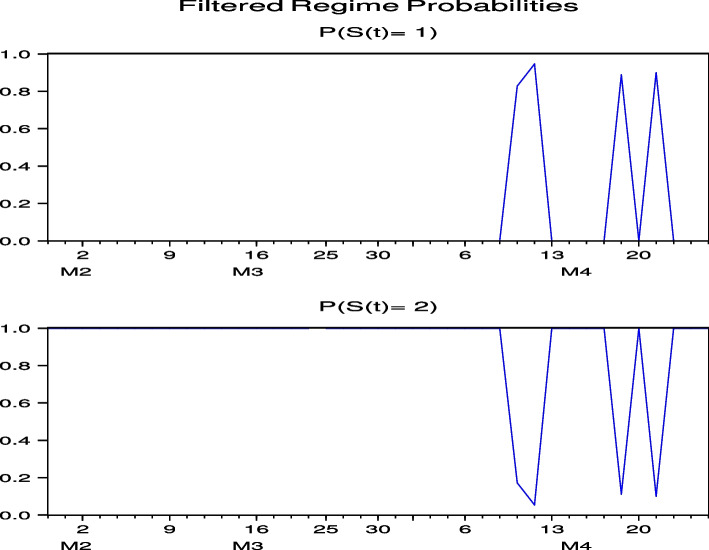
Regime probabilities for SB. A short-term sample

**Fig. 50:**
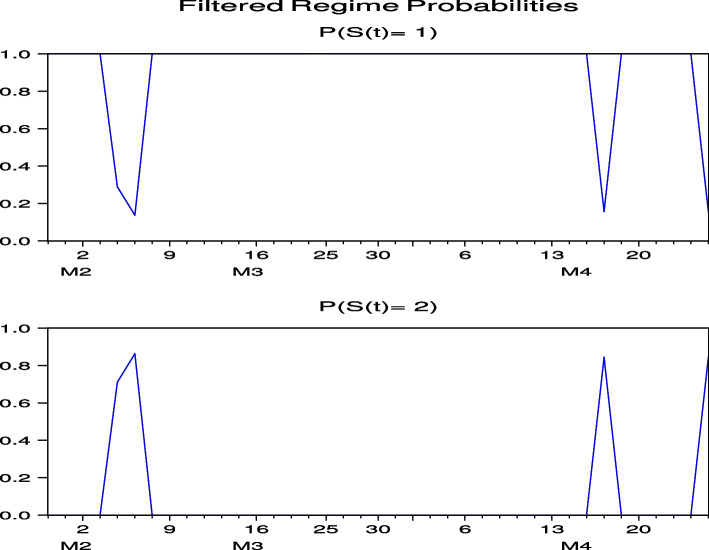
Regime probabilities for KRT. A short-term sample

## Data Availability

The stock price data employed in this study are available from Investing.com. The data of COVID-19 cases of Japan and the US are obtained from Ministry of Health, Labour and Welfare in Japan and Our World in Data.

## Appendix 1: Figures of regime probabilities

See Figs. 19, 20, 21, 22, 23, 24, 25, 26, 27, 28, 29, 30, 31, 32, 33, 34, 35, 36, 37, 38, 39, 40, 41, 42 and 43.

## Appendix 2: A short-term analysis for Japanese hotel stocks

The results for FJT, IMP, RYL, KYT, OL, SB, and KRT are presented in Table 14 and Figs. 44, 45, 46, 47, 48, 49 and 50, respectively. Using data from a short period, we obtained statistically significant and negative $\lambda _1$ or $\lambda _2$, except for RYL, which is consistent with the results of Anguera-Torrell et al. (2021). In addition, there were many cases in which the transition probabilities of $\delta _{1}$ and/or $\delta _{2}$ were not statistically significant. This is completely different from the results of our analyses using relatively long-term sample period data, as shown in Figs. 44, 46, 47, 48, 49 and 50, where we did not clearly obtain the regime probabilities. These results indicate that the short-term analysis focusing only on the first wave of COVID-19 is more affected by the speed of COVID-19 infection and does not capture regime-switching volatility well.

**Table 14:** Direct model parameter estimation for Japanese hotel stocks: a short-term sample immediately before and after COVID-19

	Regime 1			Regime 2			T-Matrix	
	*C*_1_	*λ*_1_	*k*_1_	*C*_2_	*λ*_2_	*k*_2_	*δ*_1_	*δ*_2_
FJT	**3.228**E−**03**	− **1.350**E−**04**	**− 6.536**	− 1.243E−02	2.760E−06	**− 3.111**	− 0.516	**− 2.167**
IMP	− 2.239E−03	2.190E−05	− **2.731**	1.163E−03	− **1.320**E−**04**	− **4.621**	**1.723**	− **1.362**
RYL	− **2.324**E−**03**	**5.530**E−**05**	− **5.975**	− 5.413E−03	− 4.990E−05	− **3.104**	− 20.338	− 0.428
KYT	**1.021**E−**02**	− **4.150**E−**05**	− **9.027**	− 8.209E−03	− 4.590E−05	− **3.143**	− 21.407	− **2.444**
OL	3.114E−03	− 4.710E−05	− **3.373**	**6.772**E−**03**	− **8.270**E−**05**	− **6.753**	**1.667**	0.942
SB	− **6.951**E−**03**	− **1.240**E−**05**	− **8.331**	− 6.558E−03	− 3.710E−05	− **3.467**	− 1.205	− **2.484**
KRT	− **1.626**E−**02**	5.280E−05	− **2.976**	− **3.888**E−**03**	− **1.400**E−**04**	− **7.234**	**2.581**	0.937

Note that the bold numbers represent statistical significance at 5$\%$. We obtain statistically significant and negative $\lambda _1$ or $\lambda _2$, representing the impact of changes in the number of COVID-19 cases on hotel stock prices in Regime 1 or 2, respectively, except for RYL. In addition, there were a number of cases where the transition probabilities of $\delta _{1}$ and/or $\delta _{2}$ were not statistically significant

## Abbreviations

CHH: Choice Hotels International, Inc.
FJT: Fujita Kanko, Inc.
FY: Fiscal year
H: Hyatt Hotels Corporation
HLT: Hilton Worldwide Holdings, Inc.
IHG: InterContinental Hotels Group
IMP: Imperial Hotel, Ltd.
KRT: Kyoritsu Maintenance Co., Ltd.
KYT: The Kyoto Hotel, Ltd.
MAR: Marriott International, Inc.
MC: Market capitalization
MGM: MGM Resorts International
NKY: Nikkei 225
OL: Oriental Land Co., Ltd.
PPE: Property, plant, and equipment
RYL: The Royal Hotel, Ltd.
SB: Seibu Holdings, Inc.
SPX: S&P 500
WH: Wyndham Hotels & Resorts, Inc.

## Footnotes

1 Travel balance is the difference between international tourism receipts (inbound) and international tourism expenditure (outbound).

2 Many researchers have also studied the impact of exogenous risks on travel and tourism industry (e.g., Athari et al. 2021, 2023).

3 Although IHG is a multinational hotel company based in the UK, IHG generates the largest share of its revenue in the Americas, including the US, its largest hotel market. Therefore, we analyze IHG as a US hotel stock in the sense that it is affected by COVID-19 in the US.

4 One might think that the selection of the sample by MC would affect the results of this paper. Therefore, we compute the probability that the treatment is 1 (i.e., belongs to Japan), conditional on the observed market capitalization. Note that we assume that the exchange rate is 110 USD/yen. The resulting propensity scores based on a logistic regression model for $\text {MC}^{0.4}$ with the statistically significant coefficient for $\text {MC}^{0.4}$ are two categories: between 0.8 and 0.4 and 0.1, i.e., between 0.8 and 0.4 for KYT, RYL, FJT, IMP, KRT, SB, CHH, WH, H, IHG, and MGM; 0.1 for HLT, MAR, and OL, respectively. Thus, we can safely say that both categories include Japanese and US hotels. It means that we can estimate the average Japan-US effect as a first-order approximation by comparing the treatment (Japan) and control (US) groups with similar propensity scores.

5 One might also think that this paper does not appear that all possible hospitality firms for the US and Japan are included in the analysis. For instance, Clark et al. (2021) have 62 hospitality firms from Japan in their analysis. However, this study does not deal with the hospitality industry as a whole, but focuses specifically on the hotel industry. The 154 hospitality companies in Clark et al. (2021) deal with restaurants, hotels, and casinos, of which only 41 (26.6 $\%$) are hotels. Thus, 62 Japanese hospitality companies are not our target. In addition, taking into account that these 41 hotel companies are in 23 countries, the results of the analysis using 7 companies from Japan and 7 from the US may represent the impact of COVID-19 on Japanese and US hotel stocks, respectively as a first-order approximation.

6 Japanese data start from January 4, 2019, because of the national holidays. The data source is the same as that of Anguera-Torrell et al. (2021), while our data period is longer than that of Anguera-Torrell et al. (2021).

7 RYL, KYT, SB, CHH, and HLT prices are positively skewed, while the other asset prices are negatively skewed.

8 We also obtained similar results in the ADF tests without structural breaks.

9 Although we could not find a causal relationship between the US hotels’ stock price returns and the daily changes in the number of infected people, we cannot rule out the possibility that it is due to macro explanatory factors. For example, in terms of international travel arrivals, the US had 165,478,000 in 2019 and 45,037,000 in 2020, only 27$\%$ of the previous year, while Japan had 31,881,000 in 2019 and 4,115,799.75 in 2020, only 13$\%$ of the previous year from the website of Our World in Data. The impact of COVID-19 on the US was minor compared to Japan. Therefore, the information on the number of arrivals in international travel may have an impact on the returns of hotel stocks from a long-term perspective, such as calendar years. However, from the perspective of hotel stock investors and portfolio managers who trade daily, information on international travel arrivals is not available daily. From the standpoint of data availability, we use the number of infected people as an indicator to capture COVID-19 in a first-order approximation.

10 When examining the impact factor on hotel stock prices, one might consider the COVID-19 policy on hotel availability to be significant. However, this information on the policy can be qualitative and treating it quantitatively as an explanatory variable involves arbitrariness. In addition, it would be difficult for the ordinary investors to obtain the information of the availability on a daily basis. For the impact on hotel stock prices, which are traded on a daily basis, a variable that captures the status of COVID-19 and that is available on a daily basis would be appropriate as an explanatory variable. Therefore, the number of infected persons is used as the impact of COVID-19 in this analysis. In addition, although one might question the relevance of past COVID-19 cases to hotel stock prices, which are expected financial outcomes in the future, as a backtesting this analysis examines historically whether the current stock price has moved due to changes in the number of infections one point in time ago.

11 Note that we confirmed a geometric Brownian motion model of asset prices in Eq. (1) because we found from the empirical evidence of the existence of the unit root of the log prices in “Econometric analysis” section.

12 From the perspective of ex-post analysis, i.e., consistency between the information used for parameter estimation and the information used for regime probabilities, it is considered necessary to present smoothed regime probabilities. However, for information that is unknown to humans, such as the spread of COVID-19, the calculation of regime probabilities based on all the given information is inconsistent with reality. Therefore, regime probabilities using information up to each point in time, i.e., filtered regime probabilities, are considered to be more appropriate. In addition, since the results of the smoothed regime probability are discontinuous, we adopted the filtered regime probability.

13 OL differs from the other Japanese hotel stocks in that the share of the revenue from the hotel business in its operating segments is lower, which may explain why the behavior of OL’s regime probabilities differs from that of the other Japanese hotel stocks.

14 We cannot select the order of the regimes because it is automatically selected.

15 Note that because the data used in the MS-GARCH model and the model proposed in this study differ, comparisons using log-likelihood or AIC/SIC are not possible.
